# Efficacy and safety of antibiotic regimens for *Helicobacter pylori* eradication: a systematic review and meta-analysis

**DOI:** 10.3389/fmed.2026.1780052

**Published:** 2026-04-07

**Authors:** Yinhua Liao, Hu Liu, Yibin Zhou, Zhenyu Wang

**Affiliations:** 1 Bengbu Medical University, Bengbu, China; 2 Anhui Provincial Cancer Hospital, Hefei, China; 3 Zhejiang Chinese Medical University, Hangzhou, China; 4 Medical Department, Shaoxing University, Shaoxing, China

**Keywords:** adverse events, eradication therapy, *Helicobacter pylori*, meta-analysis, treatment regimens

## Abstract

**Background:**

Helicobacter pylori infection, a global health concern, is treated with various antibiotic regimens, but rising resistance causes variable outcomes. This meta-analysis evaluated the efficacy and safety of different eradication regimens. Methods PubMed, Embase, and Web of Science were searched from inception to October 2025. Randomized and observational studies reporting eradication rates or adverse events were included. Pooled estimates were calculated using random-effects models with subgroup analyses and meta-regression. Results Fifty-seven studies (19,941 participants, 127 arms) were included. The pooled eradication rate was 83.0% (95% CI: 81.4%–84.5%; I²=87.3%). Egger's and Begg's tests indicated publication bias (both *P*<0.001); trim-and-fill adjusted rate was 78.1%. Subgroup analyses showed sequential (84.3%), quadruple (83.3%), and dual therapy (83.8%) achieved slightly higher rates than triple therapy (79.2%). PCAB-based regimens outperformed PPI-based (85.3% vs. 81.6%). Ten-day regimens yielded higher rates (85.9%) than 7-day (79.8%) or 14-day (82.7%). Regimens with three antibiotics (86.9%) or nitroimidazoles (84.8%) showed improved efficacy. Meta-regression identified publication year, continent, study design, and acid suppressant as heterogeneity sources (all *P*<0.01). For safety (54 studies, 119 arms, 5,202 events), pooled adverse event incidence was 26.1% (95% CI: 21.0%–31.9%; I²=95.3%). Sequential therapy had the highest incidence (54.1%), dual therapy the lowest (15.8%). Regimens with three antibiotics (52.6%), macrolides (29.0%), or nitroimidazoles (37.1%) were associated with more adverse events. Conclusions Eradication regimens vary in efficacy and safety; treatment selection should balance these factors. Findings require confirmation due to high heterogeneity and between-study comparisons.

**Systematic review registration:**

CRD420251207695, https://www.crd.york.ac.uk/PROSPERO/view/CRD420251207695.

## 1 Introduction

*Helicobacter pylori (H. pylori)* is a microaerophilic, Gram-negative, spiral-shaped bacterium that colonizes the human gastric mucosa. Its helical morphology and multiple flagella enable rapid movement through highly viscous gastric mucus, facilitating evasion from gastric emptying. Concurrently, its abundant urease activity hydrolyzes urea to produce ammonia, forming an “ammonia cloud” that neutralizes gastric acid in the bacterial microenvironment, thereby permitting survival in the hostile acidic environment of the stomach (1–3). These adaptive mechanisms confer upon *H. pylori* the capacity for persistent gastric colonization, establishing it as one of the most successful human pathogens known.

Epidemiological data indicate that approximately 44–50% of the global population is infected with *H. pylori*, with rates exceeding 70% in some regions; infection prevalence remains notably high in East Asia (4, 5). Consequently, improving eradication rates while mitigating antimicrobial resistance and associated complications represents a critical objective in the prevention and management of gastrointestinal diseases. Currently, eradication regimens recommended by the American College of Gastroenterology primarily include triple therapy, quadruple therapy (bismuth-containing and non-bismuth), sequential therapy, and hybrid therapy, typically comprising two to three antibiotics combined with an acid suppressant (proton pump inhibitor [PPI] or potassium-competitive acid blocker [PCAB]) (6). In response to rising antibiotic resistance, various countries have increasingly adopted strategies such as bismuth-containing quadruple therapy, high-dose PPI, or PCAB-based regimens to enhance eradication efficacy (7). However, the clinical effectiveness of these regimens varies considerably: reported eradication rates range from below 70% to over 90%, influenced by factors including regional resistance patterns, antibiotic combinations, treatment duration, and the choice and intensity of acid suppression (8–10). The incidence of adverse events also differs substantially, affecting 20–40% of patients in some regimens, particularly those containing nitroimidazoles, macrolides, or involving multiple antibiotics (11, 12).

It is noteworthy that although several meta-analyses have recently evaluated the efficacy of *H. pylori* eradication regimens, most studies possess certain limitations. First, many analyses focus narrowly on single regimen comparisons (e.g., bismuth quadruple therapy versus traditional triple therapy) or specific drug classes (e.g., comparisons between PPIs and PCABs), lacking a broad comparison across multiple mainstream therapeutic options (13–15). Second, some meta-analyses were published relatively early and did not incorporate novel regimens that have become widely adopted in recent years, such as PCAB-containing therapies or high-dose dual therapy (16–19). Third, prior studies have predominantly concentrated on eradication rates, with relatively limited systematic evaluation of adverse events, particularly lacking in-depth analysis of adverse event severity grading (20, 21). To address these research gaps, the present study offers several extensions and innovations. Firstly, we systematically included the latest clinical studies available up to 2025, encompassing 22 antibiotic combinations across regimens including triple, quadruple, sequential, and dual therapies (both bismuth-containing and bismuth-free), thereby providing a comprehensive efficacy profile of currently clinically accessible regimens. Secondly, alongside assessing eradication rates, we concurrently analyzed the overall incidence of adverse events, severity grading (mild, moderate, severe, or intolerable), and associated influencing factors, aiming to furnish evidence for clinical decision-making that balances efficacy and safety. Thirdly, through multidimensional subgroup analyses and meta-regression, we systematically explored the impact of variables such as dosage intensity, number and class of antibiotics, treatment duration, and type of acid suppression on both efficacy and safety. Furthermore, we employed the trim-and-fill method to quantify the effect of publication bias on pooled effect sizes, thereby enhancing the robustness and reliability of our findings.

Therefore, this study aims to construct a cross-regimen “selection map” to address a fundamental clinical question: among the multitude of available regimens, how can clinicians make an initial evidence-based choice based on the balance between efficacy and safety? The results are intended to complement the existing literature, which predominantly focuses on optimizing individual regimens, and to contribute to advancing more individualized and balanced clinical decision-making.

## 2 Methods

This study was designed and reported in accordance with the PRISMA 2020 guidelines (22). The protocol was registered with the International Prospective Register of Systematic Reviews (PROSPERO; registration number: CRD420251207695). Before the registration of the plan, we conducted a preliminary literature scope definition (preliminary scoping) to assist in refining the research questions and search strategies; on this basis, the research plan was finalized and registered. The formal and complete systematic literature search, literature screening, data extraction, and all analysis work were carried out strictly in accordance with the pre-registered plan, and were completed after the registration in November 2025. The search time limit covered the period from the establishment of the database to October 2025, aiming to include all the literature published before that date. Throughout the entire research process, the core content of the research plan (including inclusion criteria, exclusion criteria, outcome indicators, and subgroup analysis plans) did not undergo any changes.

### 2.1 Search strategy

Following the PRISMA 2020 guidelines, a systematic and comprehensive literature search strategy was developed and executed. The PubMed, Embase, and Web of Science (WOS) databases were searched from their inception to October 2025. The search strategy combined controlled vocabulary terms (MeSH/Emtree) and free-text keywords covering core concepts such as “*Helicobacter pylori*,” “eradication therapy,” “antibiotic regimens,” and “adverse events.” The detailed search strategy is provided in Supplementary Table 1.

### 2.2 Inclusion and exclusion criteria

The inclusion criteria for this study were as follows: (1) studies involving participants diagnosed with *H. pylori* infection using standard methods (e.g., urea breath test, rapid urease test, stool antigen test, histology); (2) studies that clearly reported the complete components of the eradication regimen, including the specific names (generic) of all antibiotics and acid suppressants, the single dose, the daily dosing frequency, and the total treatment duration (in days); (3) studies that reported outcomes related to eradication rates and adverse events, with the primary outcome being the *H. pylori* eradication rate assessed after treatment completion, and secondary outcomes including the overall incidence of adverse events (proportion of patients experiencing at least one adverse event), the incidence of adverse events by severity grade (mild, moderate, severe), and the rate of treatment discontinuation due to adverse events; (4) eligible study types included randomized controlled trials, non-randomized controlled studies, and prospective or retrospective cohort studies.

The exclusion criteria were as follows: (1) publication types such as reviews, systematic reviews, *in vitro* studies, mechanistic studies, meta-analyses, case reports, editorials, letters, and conference abstracts; (2) studies focusing on populations without *H. pylori* infection (e.g., patients with other gastrointestinal diseases only); (3) interventions other than antibiotic-based eradication therapy (e.g., probiotics alone, traditional Chinese medicine, surgery); (4) studies where the intervention regimen included probiotics, other functional foods, or herbal medicines as a core component; (5) studies where eradication efficacy was assessed earlier than four weeks after treatment completion; (6) non-English language publications.

### 2.3 Data extraction

Data extraction was performed independently by two reviewers using a standardized data extraction form. Disagreements were resolved through discussion or by adjudication of a third reviewer. The following data were extracted from each included study: (1) study information: authors, publication year, country/region, and study design type; (2) participant characteristics: number of participants in the intention-to-treat (ITT) population; number of participants in the per-protocol (PP) population was also extracted if reported; population type (general infected population, population with previous eradication failure, population with gastrointestinal symptoms) was recorded; (3) intervention details: specific antibiotic regimen (drug combination), antibiotic classes, number of antibiotics included in the regimen, dosage intensity (standard dose or high dose), treatment duration (7, 10, or 14 days), bismuth-containing status (yes/no), acid suppression type (PPI or PCAB), and regimen type (dual, triple, quadruple, sequential, or concomitant therapy); (4) study outcomes: *H. pylori* eradication rate (ITT and PP), total number of participants experiencing adverse events, and numbers of participants experiencing intolerable adverse events, as well as mild, moderate, and severe adverse events (if reported).

### 2.4 Handling of multi-arm studies

This study constitutes a single-arm meta-analysis aimed at pooling eradication rates across different treatment regimens, rather than comparing effect sizes between intervention and control groups. In multi-arm randomized controlled trials, participants in different treatment arms are randomized independently, precluding the issue of shared patient populations. To comprehensively incorporate information from all eligible treatment regimens, we retained each treatment arm from multi-arm studies as an independent data point. Concurrently, sensitivity analyses (excluding one study at a time) were conducted to assess whether any single multi-arm study exerted undue influence on the pooled effect sizes.

### 2.5 Subgroup classification

To explore potential sources of heterogeneity in eradication rates and adverse event profiles, predefined subgroup analyses were conducted. Based on treatment regimen characteristics, each study arm was categorized according to the following variables: (1) Dosage intensity: Standard dose was defined as antibiotic and acid suppressant dosages and frequencies consistent with guideline recommendations; high dose was defined as regimens with a total daily dose of amoxicillin exceeding 3.0 g, or regimens explicitly described as “high-dose” by the original study authors. (2) Treatment duration: 7 days, 10 days, or 14 days. (3) Bismuth-containing status: Bismuth-containing regimen (yes) or non-bismuth regimen (no). (4) Acid suppression type: PPI-based regimen or PCAB-based regimen. (5) Antibiotic classes: Categorization based on the antibiotics included in the regimen: *β*-lactams (e.g., amoxicillin), macrolides (e.g., clarithromycin), fluoroquinolones (e.g., levofloxacin, moxifloxacin, sitafloxacin), tetracyclines (e.g., doxycycline, minocycline), and nitroimidazoles (e.g., metronidazole, tinidazole). (6) Number of antibiotics: 1, 2, or 3. (7) Regimen type: Dual, triple, quadruple, or sequential therapy. (8) Study population characteristics: General infected population, population with previous eradication failure, or population with gastrointestinal symptoms.

### 2.6 Quality assessment

Two reviewers independently assessed the quality of the included studies. For randomized controlled trials, the Cochrane Risk of Bias tool version 2.0 was used independently by two reviewers. This tool evaluates bias across five domains: (1) bias arising from the randomization process; (2) bias due to deviations from intended interventions; (3) bias due to missing outcome data; (4) bias in measurement of the outcome; and (5) bias in selection of the reported result. The risk of bias for each domain was judged as ‘low risk,’ ‘some concerns,’ or ‘high risk.’ Any disagreements were resolved through discussion or by arbitration of a third reviewer (23).

For non-randomized or observational studies, quality was assessed using the Newcastle–Ottawa Scale (NOS) (24). Both case–control and cohort studies were scored across three major categories: selection, comparability, and exposure/outcome, with a total score ranging from 0 to 9. For case–control studies, the criteria were as follows: Selection (maximum 4 points: adequacy of case definition, representativeness of cases, selection of controls, definition of controls); Comparability (maximum 2 points: whether cases and controls were matched or adjusted for major confounding factors in the design or statistical analysis); Exposure (maximum 3 points: ascertainment of exposure, same method of ascertainment for cases and controls, non-response rate). For cohort studies, the criteria were: Selection (maximum 4 points: representativeness of the exposed cohort, selection of the non-exposed cohort, ascertainment of exposure, demonstration that outcome of interest was not present at start of study); Comparability (maximum 2 points: whether cohorts were matched or adjusted for major confounding factors in the design or analysis); Outcome (maximum 3 points: assessment of outcome, adequacy of follow-up length, adequacy of follow-up). Studies scoring 7–9 were considered high quality (low risk of bias), 4–6 moderate quality (some concerns regarding bias), and 0–3 low quality (high risk of bias).

### 2.7 Statistical analysis

In this study, eradication rates and adverse events were treated as two distinct outcome variables and analyzed separately. Given the anticipated heterogeneity across studies, a random-effects model was employed to calculate pooled effect sizes for all analyses. Eradication rates were expressed as relative risks (RR) with 95% confidence intervals (CI). For adverse events, the overall incidence was pooled for meta-analysis. Data on mild, moderate, severe, and intolerable adverse events, as reported in individual studies, were utilized for descriptive statistics.

Heterogeneity between studies was assessed using the Cochran Q test and the I^2^ statistic, with I^2^ > 50% considered indicative of substantial heterogeneity. Publication bias was evaluated using Egger’s regression test and Begg’s rank correlation test, supplemented by visual inspection of funnel plots.

To explore potential sources of heterogeneity, several predefined subgroup analyses were conducted. A random-effects model was used to generate pooled estimates within each subgroup. Meta-regression analyses were performed to examine whether study-level covariates (e.g., treatment duration, dosage intensity, antibiotic classes, bismuth use, geographic region, publication year) contributed to heterogeneity. Sensitivity analyses were conducted by sequentially excluding individual studies to assess the robustness of the pooled results and to ensure that no single study exerted undue influence on the overall estimates.

All statistical analyses were performed using Python (version 2024.3.1) and R software (version 4.4.1). All statistical tests were two-sided, and a *p*-value < 0.05 was considered statistically significant.

## 3 Results

### 3.1 Literature search

**Figure 1:**
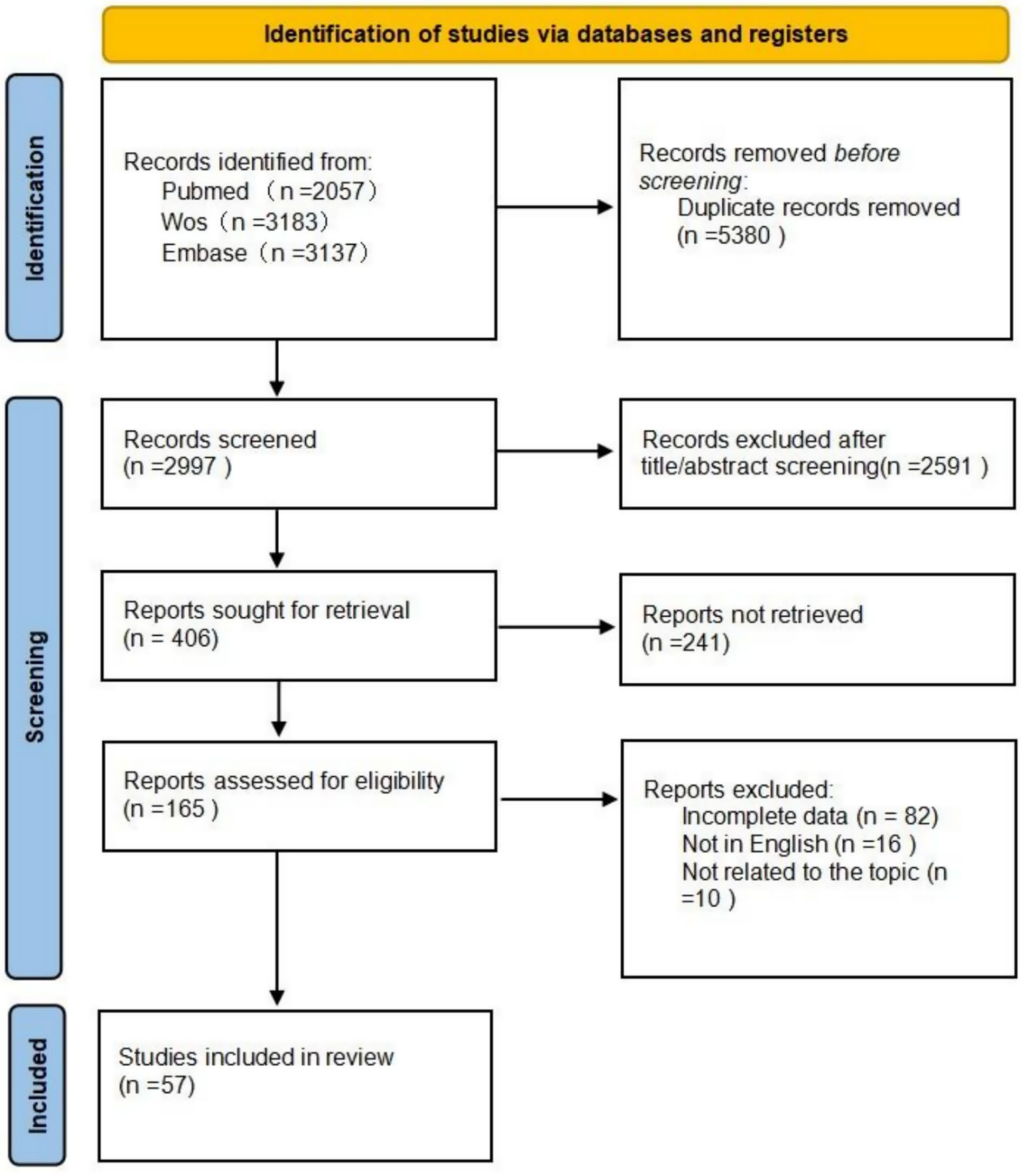
PRISMA 2020 flow diagram of study selection process.

The study selection process is presented in the PRISMA flow diagram (Figure 1). The initial search yielded 8,377 records. After removing 5,380 duplicates, 2,997 records remained for title and abstract screening, of which 2,591 were excluded as irrelevant to the topic. Full-text articles were sought for 408 potentially eligible records, but 241 could not be retrieved. A total of 165 articles were assessed in full text for eligibility. Following the exclusion of 108 articles, 57 studies were ultimately included in this review. Reasons for exclusion included incomplete or unavailable data (82 articles), non-English language publications (16 articles), and irrelevance to the research topic upon full-text review (10 articles).

### 3.2 Characteristics of included studies

**Figure 2:**
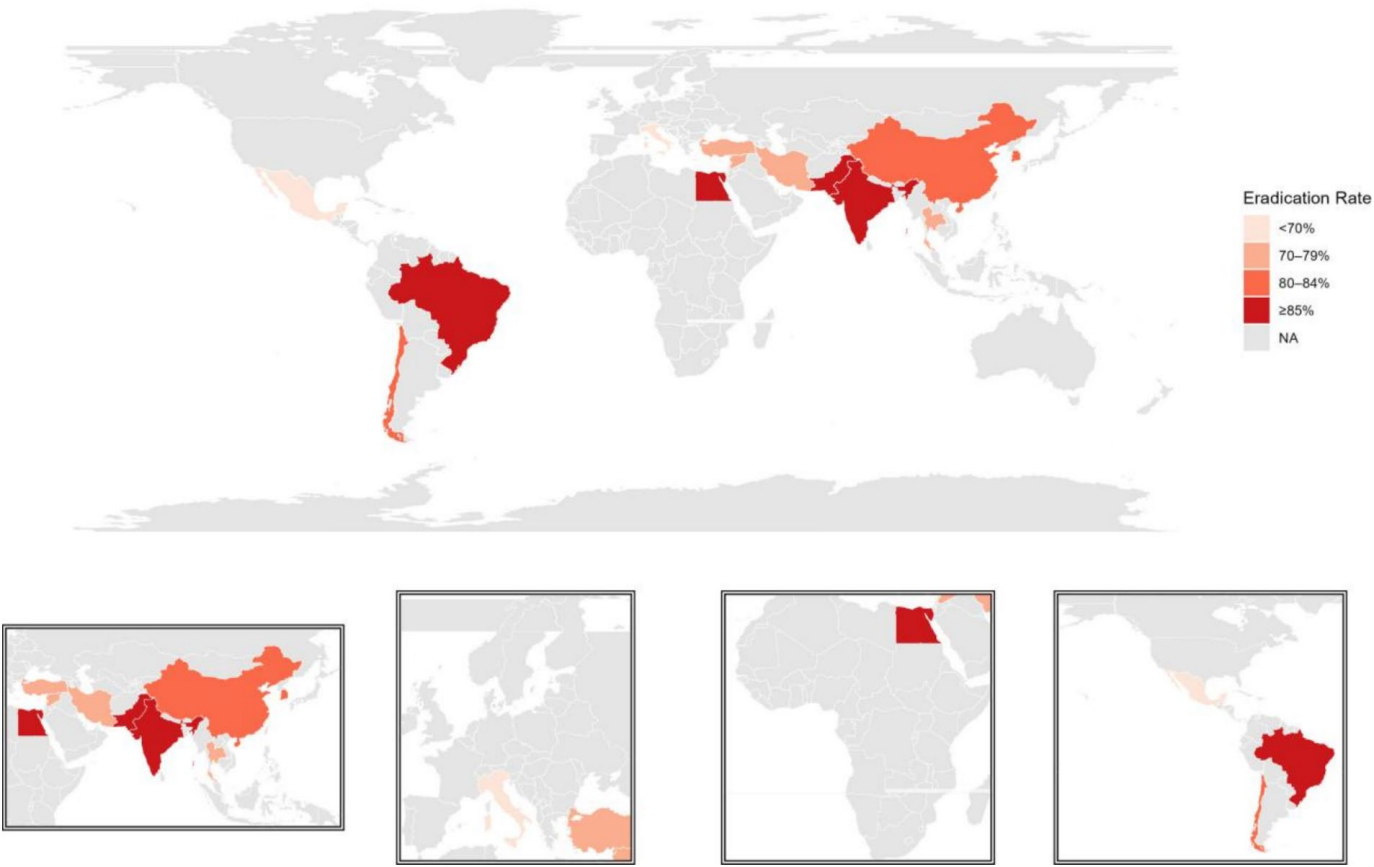
Global distribution map of *Helicobacter pylori* (Choropleth map) and local magnified map (Inset map).

A total of 57 studies, comprising 127 treatment arms, were included in this review, with publication dates ranging from 2003 to 2025. The included studies consisted primarily of randomized controlled trials, along with several non-randomized controlled studies, and the overall methodological quality was rated as moderate to high. The studies originated from 14 countries, with publications and sample sizes concentrated predominantly in Asia (mainly China). The global distribution of studies is illustrated using a choropleth map with an inset map for regional detail (Figure 2). These studies systematically evaluated a wide range of *H. pylori* eradication regimens, demonstrating substantial heterogeneity in antibiotic combinations, dosage intensity, treatment duration, acid suppressant type, and population characteristics.

#### 3.2.1 Antibiotic regimens

A total of 22 distinct antibiotic regimen types were identified among the included studies. Regimens centered on amoxicillin-based combinations were predominant, accounting for 54.5% (12/22) of all regimen types. The most frequently observed antibiotic combination was amoxicillin plus clarithromycin, representing 18.2% (4/22) of regimen types. The remaining regimens primarily consisted of tetracycline combined with metronidazole or furazolidone (4/22, 18.2%), as well as retreatment or rescue regimens containing fluoroquinolones (e.g., levofloxacin, moxifloxacin, sitafloxacin, ofloxacin), which comprised 31.8% (7/22) of regimen types. Among the included treatment arms, non-bismuth-containing regimens were more common (75/127, 59.5%), while bismuth-containing regimens (51/127, 40.2%) were primarily employed in the context of intensified treatment backgrounds. Regarding treatment duration, 14-day regimens were the most prevalent (94/127, 74.2%). Seven-day regimens (21/127, 16.5%) were predominantly observed in earlier studies or those involving fluoroquinolone-based therapies, whereas 10-day regimens (11/127, 8.7%) mainly appeared in modern protocols combining PCAB with high-dose amoxicillin. Concerning acid suppression strategy, PPI-based regimens constituted the majority (93/127, 73.2%). PCAB-based regimens (33/127, 26.0%) were notably concentrated in studies published after 2022, particularly those originating from China. In terms of dosage, standard-dose regimens accounted for the majority (101/127, 79.5%), while high-dose regimens comprised 19.7% (25/127) of treatment arms; the latter were almost exclusively centered on high-dose amoxicillin. Notably, the general infected population was the primary focus of the vast majority of studies (109/127, 85.8%).

#### 3.2.2 Adverse events

Among the 54 included studies reporting adverse event data (three studies were excluded as they reported the number of events rather than the number of participants experiencing events), all reported the total number of participants experiencing adverse events, with a cumulative total of 6,088 participants experiencing at least one adverse event. Of these studies, 17 further reported the number of participants experiencing intolerable adverse events, totaling 56 cases. Sixteen studies reported mild adverse events, encompassing 1,218 cases (approximately 20.00% of all adverse events). Fifteen studies reported moderate adverse events, comprising 216 cases (approximately 3.5%). Ten studies reported severe adverse events, totaling 74 cases (approximately 1.2%). Overall, the majority of adverse events were mild or moderate in severity, with severe and intolerable adverse events being relatively uncommon.

### 3.3 Quality assessment results

Of the 57 included studies, 44 were randomized controlled trials and 13 were non-randomized or observational studies. According to the RoB 2 assessment, most included randomized controlled trials were judged as low risk of bias across domains including random sequence generation, allocation concealment, blinding of participants and personnel (performance bias), blinding of outcome assessment (detection bias), and selective reporting. This suggests a generally sound methodological quality in the overall study design. However, some studies presented unclear risk in the domain of incomplete outcome data. Deductions in quality scores for most randomized controlled trials primarily stemmed from incomplete reporting of outcome measures; for instance, some studies reported only *H. pylori* eradication rates based on intention-to-treat (ITT) analysis without concurrently presenting per-protocol (PP) analysis results. Although these issues may introduce some degree of information bias, their overall impact on the internal validity of the studies is considered limited (Supplementary Figures 1, 2).

The 13 non-randomized studies were assessed using the Newcastle–Ottawa Scale, with scores ranging from 7 to 8 and a median score of 7. Primary limitations contributing to score deductions in non-randomized studies included restricted sample sources (e.g., single-center recruitment) and inadequate adjustment for potential confounding factors. While these limitations are commonly encountered in real-world studies, the overall scores remained within the high-quality range (Supplementary Table 2).

In summary, the overall quality of the literature included in this review was satisfactory. Most studies exhibited only limited methodological limitations, and the risk of bias exerting a substantial systematic influence on the primary outcomes (eradication rates and adverse event incidence) is considered low.

### 3.4 Analysis of eradication rates

#### 3.4.1 Meta-analysis of overall eradication rate and publication Bias assessment

**Figure 3:**
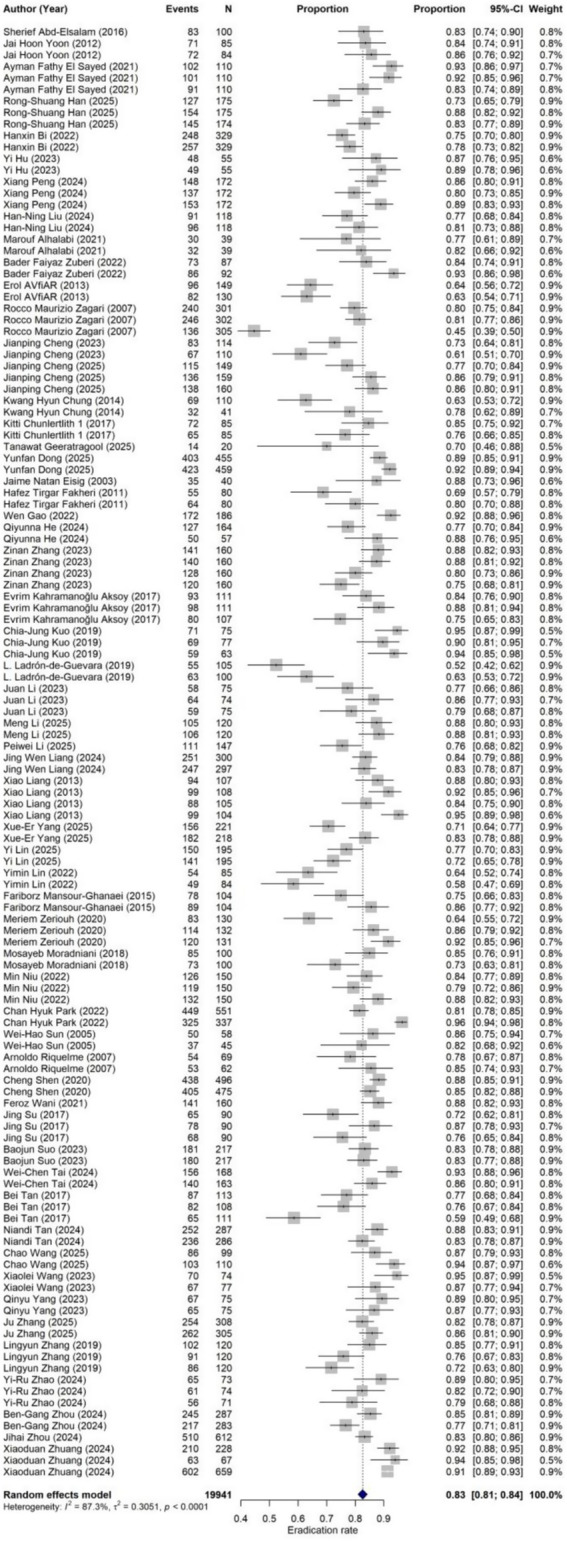
Forest plot of eradication rate.

Eradication rate data were extracted from a total of 127 treatment arms, encompassing 19,941 participants, among whom 16,387 achieved successful *H. pylori* eradication. The forest plot for the overall eradication rate is presented in Figure 3. The random-effects model demonstrated a pooled overall eradication rate of 82.64% (95% CI: 81.03 to 84.14%). Substantial heterogeneity was observed across studies (I^2^ = 87.3%; τ^2^ = 0.3051; Q = 984.02, df = 125, *p* < 0.0001), indicating considerable variability among studies in terms of population characteristics, antibiotic regimen combinations, dosage intensity, treatment duration, and type of acid suppression.

**Figure 4:**
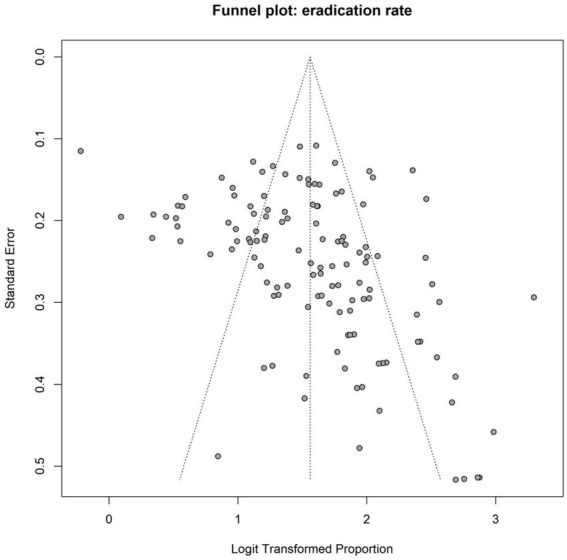
Funnel plot for eradication rates.

**Figure 5:**
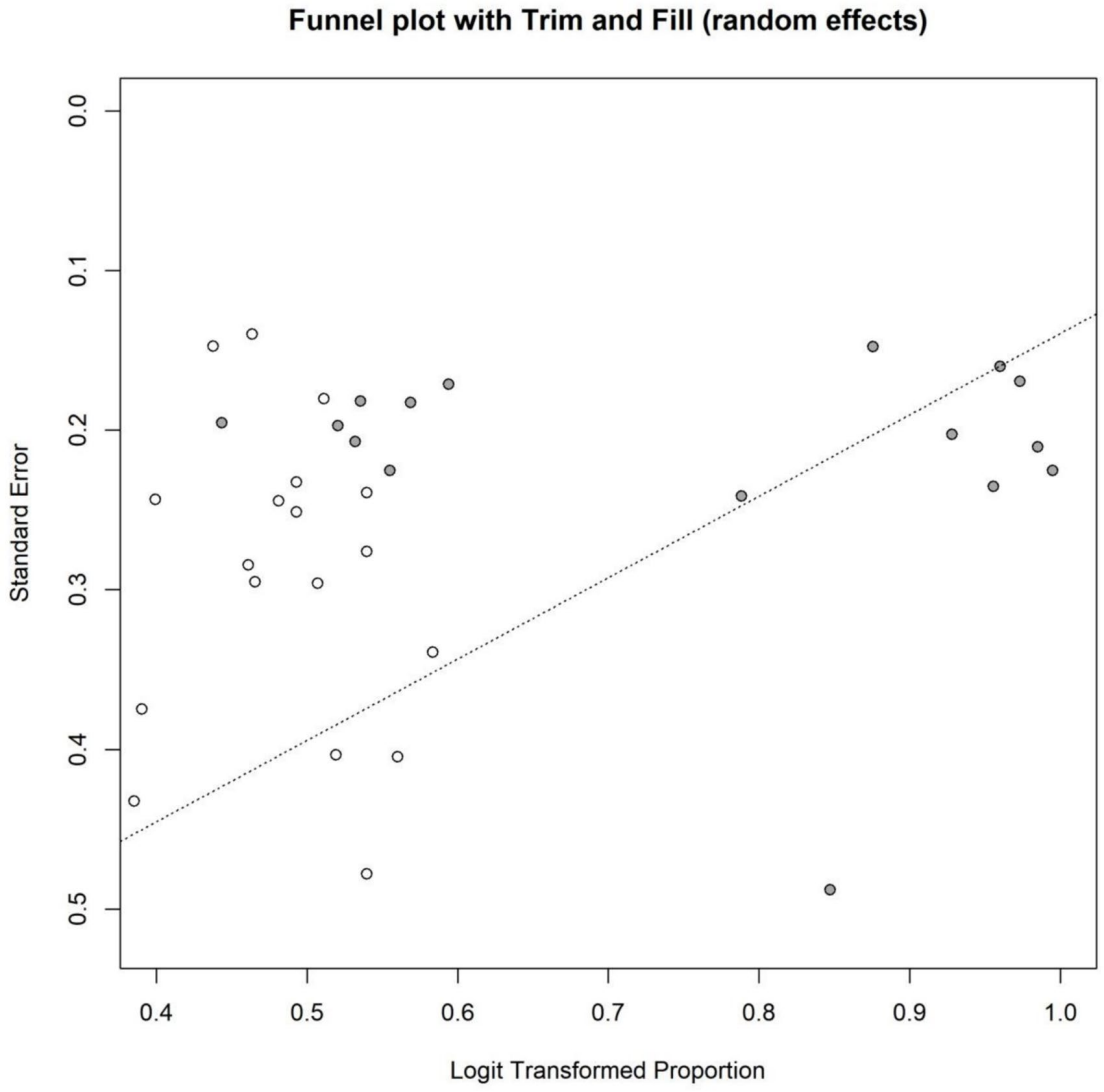
Graph of the patching method for eradication rate.

Both Egger’s test (*t* = 3.97, df = 124, *p* = 0.0001) and Begg’s test (*z* = 4.28, *p* < 0.0001) suggested the presence of significant small-study effects or publication bias. The statistical significance of both tests indicates that smaller studies reporting higher eradication rates may have been more likely to be published or included, potentially leading to an overestimation of the overall eradication rate. The funnel plot for the overall eradication rate is shown in Figure 4, further corroborating the results of Egger’s and Begg’s tests, namely the potential presence of publication bias or small-study effects. The trim-and-fill method was employed to further assess the impact of publication bias on the pooled effect size. Results indicated that funnel plot asymmetry might be attributable to 38 missing studies. After applying the trim-and-fill method, the pooled eradication rate derived from the random-effects model was 78.13% (95% CI: 75.93 to 80.17%), which was lower than the original pooled estimate of 82.64% (95% CI: 81.03 to 84.14%). This suggests that the original estimate may have overestimated the overall eradication rate due to publication bias or small-study effects. The trim-and-fill funnel plot is presented in Figure 5, visually displaying the imputed study positions and the distribution of adjusted effect sizes. This finding aligns with the conclusions of Egger’s and Begg’s tests, further confirming the presence of publication bias (Table 1).

**Table 1:** Characteristics of included studies,*Indicates studies not included in the adverse event analysis.

No.	Author (Year)	Country	Research type	ITT	Antibiotics	Dose intensity	Bismuth compound	Treatment plan course	Acid suppression type	Population characteristics	The number of eradicated cases	Total with adverse reactions
1	Sherief Abd-Elsalam (2016) (45)	Egypt	Non-RCT	100	Levofloxacin + nitazoxanide + doxycycline	Standard dose	No	14	PPI	Previously failed eradication	83	51
2	Jai Hoon Yoon (2012) (46)	Korea	RCT	85	Amoxicillin + clarithromycin	Standard dose	No	7	PPI	General population	71	19
				84	Amoxicillin + clarithromycin	Standard dose	No	14	PPI	General population	72	32
3	Ayman Fathy El Sayed (2021) (47)	Egypt	RCT	110	Amoxicillin + clarithromycin + metronidazole	Standard dose	No	14	PPI	General population	102	48
				110	Amoxicillin + clarithromycin + metronidazole	Standard dose	No	14	PPI	General population	101	58
				110	Levofloxacin + nitazoxanide + doxycycline	Standard dose	No	10	PPI	General population	91	45
4	Rong-Shuang Han (2025) (48)	China	RCT	175	Amoxicillin + clarithromycin	Standard dose	Yes	14	PPI	General population	127	52
				175	Amoxicillin + clarithromycin	Standard dose	No	14	PCAB	General population	154	48
				174	Amoxicillin + clarithromycin	Standard dose	No	10	PCAB	General population	145	42
5	Hanxin Bi (2022) (49)	China	RCT	329	Amoxicillin	High dose	No	14	PPI	Previously failed eradication	248	34
				329	Tetracycline + furazolidone	Standard dose	Yes	14	PPI	Previously failed eradication	257	81
6	Yi Hu (2023) (50)	China	RCT	55	Amoxicillin	High dose	No	14	PCAB	General population	48	54
				55	Amoxicillin	Standard dose	No	14	PCAB	General population	49	54
7	Xiang Peng (2024) (51)	China	RCT	172	Amoxicillin	High dose	No	10	PCAB	General population	148	34
				172	Amoxicillin	Standard dose	No	10	PCAB	General population	137	30
				172	Amoxicillin	High dose	No	14	PCAB	General population	153	39
8	Han-Ning Liu(2024) (52)	China	RCT	118	Amoxicillin	High dose	No	14	PCAB	General population	91	5
				118	Amoxicillin + clarithromycin	Standard dose	Yes	14	PCAB	General population	96	18
9	Niandi Tan (2024) (53)	China	RCT	287	Amoxicillin + clarithromycin	Standard dose	Yes	14	PCAB	General population	252	219
				286	Amoxicillin + clarithromycin	Standard dose	Yes	14	PPI	General population	236	222
10*	Marouf Alhalabi (2021) (54)	Syria	RCT	39	Doxycycline + tinidazole	Standard dose	Yes	14	PPI	General population	30	47
				39	Amoxicillin + levofloxacin + tinidazole	Standard dose	No	14	PPI	General population	32	34
11	Bader Faiyaz Zuberi (2022) (55)	Pakistan	RCT	87	Amoxicillin + clarithromycin	Standard dose	No	14	PPI	General population	73	33
				92	Amoxicillin	Standard dose	No	14	PCAB	General population	86	12
12*	Erol AVfiAR (2013) (56)	Turkey	RCT	149	Amoxicillin + clarithromycin	Standard dose	No	14	PPI	General population	96	191
				130	Amoxicillin + clarithromycin	Standard dose	No	14	PPI	General population	82	187
13	Rocco Maurizio Zagari (2007) (57)	Italy	RCT	301	Amoxicillin	Standard dose	No	14	PPI	General population	240	33
				302	Amoxicillin + clarithromycin	Standard dose	No	7	PPI	General population	246	42
				305	Amoxicillin + clarithromycin	Standard dose	No	14	PPI	General population	136	20
14	Jianping Cheng (2023) (58)	China	RCT	114	Amoxicillin	High dose	No	14	PPI	General population	83	31
				110	Amoxicillin + clarithromycin	Standard dose	Yes	14	PPI	General population	67	39
15	Jianping Cheng (2025) (59)	China	RCT	149	Amoxicillin + clarithromycin	Standard dose	Yes	14	PPI	General population	115	37
				159	Amoxicillin	High dose	No	14	PCAB	General population	136	24
				160	Amoxicillin + clarithromycin	Standard dose	Yes	14	PCAB	General population	138	33
16	Kwang Hyun Chung (2014) (60)	Korea	Non-RCT	110	Amoxicillin + moxifloxacin	Standard dose	No	7	PPI	Previously failed eradication	69	30
				41	Metronidazole + minocycline	High dose	Yes	7	PPI	Previously failed eradication	32	29
17	Kitti Chunlertlith (2017) (61)	Thailand	RCT	85	Amoxicillin + clarithromycin	High dose	No	14	PPI	General population	72	51
				85	Amoxicillin + clarithromycin	Standard dose	No	14	PPI	General population	65	62
18	Tanawat Geeratragool (2025) (62)	Thailand	RCT	20	Amoxicillin + sitafloxacin	Standard dose	No	7	PCAB	Previously failed eradication	14	3
19	Yunfan Dong (2025) (63)	China	RCT	455	Amoxicillin	Standard dose	No	10	PCAB	General population	403	91
				459	Amoxicillin	Standard dose	No	14	PCAB	General population	423	82
20	Jaime Natan Eisig (2003) (64)	Brazil	Non-RCT	40	Clarithromycin + metronidazole	Standard dose	No	7	PPI	General population	35	6
21	Hafez Tirgar Fakheri (2011) (65)	Iran	RCT	80	Amoxicillin + metronidazole + furazolidone	Standard dose	No	14	PPI	General population	55	78
				80	Amoxicillin + furazolidone	Standard dose	Yes	14	PPI	General population	64	80
22	Wen Gao (2022) (66)	China	Non-RCT	186	Amoxicillin	High dose	No	14	PCAB	General population	172	14
23	Qiyunna He (2024) (67)	China	RCT	164	Amoxicillin + minocycline	Standard dose	Yes	14	PCAB	General population	127	9
				57	Amoxicillin + furazolidone	Standard dose	Yes	14	PPI	General population	50	5
24	Zinan Zhang (2023) (68)	China	RCT	160	Doxycycline + furazolidone	Standard dose	No	14	PCAB	General population	141	22
				160	Amoxicillin+doxycycline	Standard dose	No	14	PCAB	General population	140	24
				160	Doxycycline + furazolidone	Standard dose	Yes	14	PPI	General population	128	26
				160	Amoxicillin+doxycycline	Standard dose	Yes	14	PPI	General population	120	23
25*	Evrim Kahramanoğlu Aksoy (2017) (69)	Turkey	RCT	111	Amoxicillin+ levofloxacin	Standard dose	Yes	14	PPI	General population	93	156
				111	Metronidazole + minocycline	High dose	Yes	14	PPI	General population	98	215
				107	Amoxicillin+ levofloxacin	Standard dose	No	14	PPI	General population	80	56
26	Chia-Jung Kuo (2019) (70)	China	Non-RCT	75	Amoxicillin + clarithromycin	Standard dose	No	7	PPI	General population	71	10
				77	Amoxicillin + clarithromycin	Standard dose	No	7	PPI	General population	69	16
				63	Amoxicillin + clarithromycin	Standard dose	No	7	PPI	General population	59	4
27	L. Ladrón-de-Guevara (2019) (71)	Mexico	RCT	105	Amoxicillin + clarithromycin	Standard dose	No	14	PPI	General population	55	86
				100	Amoxicillin+ levofloxacin	Standard dose	No	14	PPI	General population	63	68
28	Juan Li (2023) (72)	China	RCT	75	Amoxicillin	High dose	No	14	PCAB	General population	58	6
				74	Amoxicillin	High dose	No	14	PCAB	General population	64	1
				75	Amoxicillin + furazolidone	Standard dose	Yes	14	PPI	General population	59	14
29	Meng Li (2025) (73)	China	RCT	120	Minocycline	Standard dose	No	14	PCAB	General population	105	20
				120	Amoxicillin + clarithromycin	Standard dose	Yes	14	PPI	General population	106	34
30	Peiwei Li (2025) (74)	China	Non-RCT	147	Amoxicillin	Standard dose	Yes	10	PPI	General population	111	20
31	Jing Wen Liang (2024) (75)	China	Non-RCT	300	Amoxicillin	High dose	Yes	14	PCAB	General population	251	41
				297	Amoxicillin + clarithromycin	High dose	Yes	14	PPI	General population	247	85
32	Xiao Liang (2013) (76)	China	RCT	107	Metronidazole + minocycline	High dose	Yes	14	PPI	General population	94	46
				108	Tetracycline + furazolidone	High dose	Yes	14	PPI	General population	99	27
				105	Amoxicillin+tetracycline	High dose	Yes	14	PPI	General population	88	15
				104	Amoxicillin + furazolidone	High dose	Yes	14	PPI	General population	99	22
33	Xue-Er Yang (2025) (77)	China	RCT	221	Amoxicillin	High dose	No	14	PPI	General population	156	11
				218	Amoxicillin + clarithromycin	Standard dose	Yes	14	PPI	General population	182	19
34	Yi Lin (2025) (78)	China	RCT	195	Metronidazole + minocycline	Standard dose	Yes	14	PPI	General population	150	62
				195	Amoxicillin + clarithromycin	Standard dose	Yes	14	PPI	General population	141	34
35	Yimin Lin (2022) (79)	China	RCT	85	Amoxicillin	High dose	No	7	PCAB	General population	54	20
				84	Amoxicillin	High dose	No	7	PCAB	General population	49	14
36	Fariborz Mansour-Ghanaei (2015) (80)	Iran	RCT	104	Amoxicillin + clarithromycin + tinidazole	Standard dose	Yes	7	PPI	Previously failed eradication	78	55
				104	Tetracycline + metronidazole + ofloxacin	Standard dose	Yes	7	PPI	Previously failed eradication	89	26
37	Meriem Zeriouh (2020) (81)	Maldives	RCT	130	Amoxicillin	High dose	No	14	PPI	General population	83	27
				132	Amoxicillin	Standard dose	No	10	PPI	General population	114	53
				131	Amoxicillin + clarithromycin + metronidazole	Standard dose	No	10	PPI	General population	120	107
38	Mosayeb Moradniani (2018) (82)	Iran	RCT	100	Amoxicillin+ levofloxacin	Standard dose	No	7	PPI	General population	85	43
				100	Amoxicillin + clarithromycin	Standard dose	No	7	PPI	General population	73	53
39	Min Niu (2022) (83)	China	RCT	150	Amoxicillin + furazolidone	Standard dose	Yes	14	PPI	General population	126	31
				150	Amoxicillin + furazolidone	Standard dose	Yes	10	PPI	General population	119	28
				150	Amoxicillin	High dose	No	14	PPI	General population	132	12
40	Chan Hyuk Park (2022) (84)	Korea	Non-RCT	551	Amoxicillin	High dose	No	14	PPI	General population	449	160
				337	Amoxicillin + clarithromycin + metronidazole	Standard dose	No	10	PCAB	General population	325	173
41	Wei-Hao Sun (2005) (85)	China	RCT	58	Amoxicillin + clarithromycin	Standard dose	No	7	PPI	General population	50	6
		China		45	Amoxicillin + clarithromycin	Standard dose	No	7	PPI	General population	37	5
42	Arnoldo Riquelme (2007) (86)	Chile	RCT	69	Amoxicillin + clarithromycin	Standard dose	No	7	PPI	General population	54	20
				62	Amoxicillin + clarithromycin	Standard dose	No	14	PPI	General population	53	23
43	Cheng Shen (2020) (87)	China	RCT	496	Amoxicillin	High dose	No	14	PPI	General population	438	92
				475	Amoxicillin + clarithromycin	Standard dose	Yes	14	PPI	General population	405	172
44	Feroz Wani (2021) (88)	India	Non-RCT	160	Amoxicillin+nitazoxanide+moxifloxacin	Standard dose	No	14	PPI	General population	141	42
45	Jing Su (2017) (89)	China	RCT	90	Amoxicillin + clarithromycin	Standard dose	Yes	7	PPI	Digestive symptoms population	65	3
				90	Amoxicillin+ levofloxacin	Standard dose	Yes	7	PPI	Digestive symptoms population	78	1
				90	Amoxicillin+ levofloxacin	Standard dose	No	7	PPI	Digestive symptoms population	68	1
46	Baojun Suo (2023) (90)	China	RCT	217	Metronidazole + minocycline	Standard dose	Yes	14	PPI	Previously failed eradication	181	75
				217	Metronidazole + minocycline	Standard dose	Yes	14	PPI	Previously failed eradication	180	88
47	Wei-Chen Tai (2024) (91)	China	Non-RCT	168	Amoxicillin	High dose	No	14	PPI	General population	156	20
				163	Amoxicillin	High dose	No	14	PPI	General population	140	20
48	Bei Tan (2017) (92)	China	RCT	113	Amoxicillin + clarithromycin	Standard dose	No	14	PPI	General population	87	3
				108	Amoxicillin + clarithromycin	High dose	No	14	PPI	General population	82	5
				111	Amoxicillin + clarithromycin	Standard dose	No	14	PPI	General population	65	1
49	Chao Wang (2025) (93)	China	RCT	99	Amoxicillin + furazolidone	Standard dose	Yes	14	PPI	General population	86	58
				110	Amoxicillin	High dose	No	14	PPI	General population	103	10
50	Xiaolei Wang (2023) (94)	China	RCT	74	Amoxicillin	High dose	No	14	PCAB	General population	70	29
				77	Amoxicillin + clarithromycin	Standard dose	Yes	14	PPI	General population	67	61
51	Qinyu Yang (2023) (95)	China	RCT	75	Amoxicillin	High dose	No	14	PPI	Previously failed eradication	67	8
				75	Amoxicillin + clarithromycin	Standard dose	Yes	14	PPI	Previously failed eradication	65	20
52	Ju Zhang (2025) (96)	China	RCT	308	Amoxicillin	High dose	No	14	PCAB	Previously failed eradication	254	46
				305	Tetracycline + furazolidone	Standard dose	Yes	14	PPI	Previously failed eradication	262	98
53	Lingyun Zhang (2019) (97)	China	RCT	120	Amoxicillin + minocycline	Standard dose	Yes	14	PPI	General population	102	36
				120	Metronidazole + minocycline	Standard dose	Yes	14	PPI	General population	91	45
				120	Amoxicillin + clarithromycin	Standard dose	Yes	14	PCAB	General population	86	48
54	Yi-Ru Zhao (2024) (98)	China	Non-RCT	73	Amoxicillin+tetracycline	Standard dose	Yes	14	PPI	General population	65	73
				74	Amoxicillin+tetracycline	Standard dose	Yes	14	PPI	General population	61	74
				71	Amoxicillin+tetracycline	Standard dose	Yes	14	PPI	General population	56	71
55	Ben-Gang Zhou (2024) (99)	China	RCT	287	Amoxicillin	High dose	No	10	PCAB	General population	245	26
				283	Amoxicillin	High dose	No	14	PPI	General population	217	33
56	Jihai Zhou (2024) (100)	China	Non-RCT	612	Amoxicillin + clarithromycin	Standard dose	Yes	14	PCAB	General population	510	80
57	Xiaoduan Zhuang (2024) (101)	China	Non-RCT	228	Amoxicillin + clarithromycin	Standard dose	Yes	14	PPI	General population	210	60
				67	Amoxicillin + clarithromycin	Standard dose	Yes	14	PCAB	General population	63	31
				659	Amoxicillin + clarithromycin	Standard dose	Yes	14	PPI	General population	602	151

#### 3.4.2 Subgroup analysis of overall eradication rate

Following multidimensional subgroup analyses of eradication rates (Table 2), the pooled eradication rates across most subgroups ranged from 0.79 to 0.86. However, moderate to high heterogeneity persisted within subgroups (I^2^ > 70% for the majority).

**Table 2:** Subgroup analysis results of eradication rate.

Subgroup		Studies	Patients	Pooled rate	95% CI	I^2^	Tau^2^	Q	*p*-value	Prediction interval
Dose_intensity	Dose_intensity_standard	101	15,386.0	0.826	[0.808, 0.843]	87.5%	0.3345	796.83	0.0000	[0.000, 1.000]
	Dose_intensity_high	25	4,555.0	0.828	[0.794, 0.858]	87.2%	0.2730	187.19	0.0000	[0.000, 1.000]
Scheme_type	Scheme_type_triple	31	3,206.0	0.792	[0.742, 0.835]	90.4%	0.5563	311.90	0.0000	[0.000, 1.000]
	Scheme_type_sequential	8	872.0	0.843	[0.779, 0.891]	80.1%	0.2949	35.14	0.0000	[0.000, 1.000]
	Scheme_type_quadruple	54	9,042.0	0.833	[0.815, 0.850]	78.9%	0.1695	251.18	0.0000	[0.000, 1.000]
	Scheme_type_dual	33	6,821.0	0.838	[0.811, 0.862]	87.1%	0.2488	247.30	0.0000	[0.000, 1.000]
Population_characteristics	Population_characteristics_prior_failure	14	2,334.0	0.805	[0.770, 0.836]	71.9%	0.1038	46.26	0.0000	[0.072, 1.000]
	Population_characteristics_general	109	17,337.0	0.831	[0.813, 0.847]	88.4%	0.3545	929.09	0.0000	[0.000, 1.000]
	Population_characteristics_symptomatic	3	270.0	0.783	[0.686, 0.857]	65.6%	0.1301	5.82	0.0546	[0.000, 1.000]
Bismuth_compound	Bismuth_compound_no	75	11,423.0	0.827	[0.803, 0.850]	90.4%	0.4516	768.13	0.0000	[0.000, 1.000]
	Bismuth_compound_yes	51	8,518.0	0.825	[0.807, 0.842]	75.3%	0.1324	202.10	0.0000	[0.084, 1.000]
Acid_suppression	Acid_suppression_type_ppi	93	13,885.0	0.816	[0.796, 0.834]	87.3%	0.3166	725.75	0.0000	[0.000, 1.000]
	Acid_suppression_type_pcab	33	6,056.0	0.853	[0.827, 0.875]	83.3%	0.2326	191.14	0.0000	[0.000, 1.000]
Treatment_plan_course	Treatment_plan_course_14_days	94	15,842.0	0.827	[0.809, 0.845]	88.4%	0.3275	803.45	0.0000	[0.000, 1.000]
	Treatment_plan_course_7_days	21	1,832.0	0.798	[0.754, 0.836]	77.2%	0.2523	87.65	0.0000	[0.000, 1.000]
	Treatment_plan_course_10_days	11	2,267.0	0.859	[0.819, 0.891]	82.1%	0.1987	55.96	0.0000	[0.000, 1.000]
The_quantity_of_antibiotics	The_quantity_of_antibiotics_3_antibiotics	11	1,385.0	0.869	[0.811, 0.911]	85.0%	0.4523	66.59	0.0000	[0.000, 1.000]
	The_quantity_of_antibiotics_2_antibiotics	80	11,684.0	0.814	[0.793, 0.834]	87.4%	0.3278	626.44	0.0000	[0.000, 1.000]
	The_quantity_of_antibiotics_1_antibiotic	35	6,872.0	0.838	[0.812, 0.862]	86.6%	0.2522	254.09	0.0000	[0.000, 1.000]
β-lactam_antibiotics	Β-lactam_antibiotics_no_β-lactam	18	2,583.0	0.833	[0.810, 0.854]	50.3%	0.0523	34.23	0.0078	[0.322, 1.000]
	Β-lactam_antibiotics_with_*β*-lactam	108	17,358.0	0.825	[0.807, 0.842]	88.7%	0.3542	943.12	0.0000	[0.000, 1.000]
Macrolide_antibiotics	Macrolide_antibiotics_no_macrolide	77	11,944.0	0.828	[0.811, 0.843]	80.3%	0.1917	385.75	0.0000	[0.000, 1.000]
	Macrolide_antibiotics_with_macrolide	49	7,997.0	0.824	[0.791, 0.853]	91.8%	0.5010	583.60	0.0000	[0.000, 1.000]
Quinolones_antibiotics	Quinolones_antibiotics_with_quinolone	12	1,137.0	0.791	[0.733, 0.838]	77.8%	0.2368	49.61	0.0000	[0.000, 1.000]
	Quinolones_antibiotics_no_quinolone	114	18,804.0	0.830	[0.813, 0.846]	87.8%	0.3212	928.08	0.0000	[0.000, 1.000]
Tetracyclines_antibiotics	Tetracyclines_antibiotics_with_tetracycline	26	3,580.0	0.833	[0.813, 0.851]	55.4%	0.0660	56.04	0.0004	[0.284, 1.000]
	Tetracyclines_antibiotics_no_tetracycline	100	16,361.0	0.825	[0.805, 0.843]	89.2%	0.3695	919.91	0.0000	[0.000, 1.000]
Nitroimidazoles_antibiotics	Nitroimidazoles_antibiotics_with_nitroimidazole	32	4,249.0	0.848	[0.824, 0.869]	73.8%	0.1714	118.33	0.0000	[0.000, 1.000]
	Nitroimidazoles_antibiotics_no_nitroimidazole	94	15,692.0	0.819	[0.799, 0.837]	89.0%	0.3442	844.57	0.0000	[0.000, 1.000]

##### 3.4.2.1 Dosage intensity

When stratified by dosage intensity, standard-dose and high-dose regimens yielded comparable eradication rates. The standard-dose regimens, comprising 101 treatment arms with 15,386 patients, had a pooled eradication rate of 82.6% (95% CI: 80.8 to 84.3%; I^2^ = 87.5%). High-dose regimens, comprising 25 treatment arms with 4,555 patients, had a pooled eradication rate of 82.8% (95% CI: 79.4 to 85.8%; I^2^ = 87.2%), both exhibiting high heterogeneity.

##### 3.4.2.2 Regimen type

Eradication rates varied somewhat across different regimen types. Triple therapy (31 arms, 3,206 patients) showed an eradication rate of 79.2% (95% CI: 74.2 to 83.5%; I^2^ = 90.4%). Sequential therapy (8 arms, 872 patients) demonstrated a rate of 84.3% (95% CI: 77.9 to 89.1%; I^2^ = 80.1%). Quadruple therapy (54 arms, 9,042 patients) yielded a rate of 83.3% (95% CI: 81.5 to 85.0%; I^2^ = 78.9%). Dual therapy (33 arms, 6,821 patients) showed a rate of 83.8% (95% CI: 81.1 to 86.2%; I^2^ = 87.1%). Overall, the pooled eradication rates for sequential, quadruple, and dual therapies were marginally higher than those for traditional triple therapy.

##### 3.4.2.3 Population characteristics

The eradication rate was slightly lower in populations with prior eradication failure compared to the general population. Among patients with prior failure (14 arms, 2,334 patients), the pooled eradication rate was 80.5% (95% CI: 77.0 to 83.6%; I^2^ = 71.9%). In the general population (109 arms, 17,337 patients), the rate was 83.1% (95% CI: 81.3 to 84.7%; I^2^ = 88.4%). Fewer studies focused exclusively on symptomatic populations (3 arms, 270 patients), yielding an eradication rate of 78.3% (95% CI: 68.6 to 85.7%; I^2^ = 65.6%).

##### 3.4.2.4 Bismuth-containing status

Overall eradication rates were very similar between bismuth-containing and non-bismuth regimens. Non-bismuth regimens (75 arms, 11,423 patients) had a pooled eradication rate of 82.7% (95% CI: 80.3 to 85.0%; I^2^ = 90.4%). Bismuth-containing regimens (51 arms, 8,518 patients) had a rate of 82.5% (95% CI: 80.7 to 84.2%; I^2^ = 75.3%). Notably, heterogeneity was relatively lower among bismuth-containing regimens.

##### 3.4.2.5 Acid suppression type

Regimens employing PCAB demonstrated higher overall eradication rates compared to those using PPI. The PPI group (93 arms, 13,885 patients) showed an eradication rate of 81.6% (95% CI: 79.6 to 83.4%; I^2^ = 87.3%). The PCAB group (33 arms, 6,056 patients) showed a rate of 85.3% (95% CI: 82.7 to 87.5%; I^2^ = 83.3%).

##### 3.4.2.6 Treatment duration

Eradication rates varied according to treatment duration. Fourteen-day regimens (94 arms, 15,842 patients) had an eradication rate of 82.7% (95% CI: 80.9 to 84.5%; I^2^ = 88.4%). Seven-day regimens (21 arms, 1,832 patients) exhibited a lower rate of 79.8% (95% CI: 75.4 to 83.6%; I^2^ = 77.2%). Ten-day regimens (11 arms, 2,267 patients) showed the highest rate at 85.9% (95% CI: 81.9 to 89.1%; I^2^ = 82.1%).

##### 3.4.2.7 Number of antibiotics

Regimens containing three antibiotics demonstrated a relatively higher eradication rate. Regimens with three antibiotics (11 arms, 1,385 patients) had a pooled eradication rate of 86.9% (95% CI: 81.1 to 91.1%; I^2^ = 85.0%). Regimens containing two antibiotics (80 arms, 11,684 patients) showed a rate of 81.4% (95% CI: 79.3 to 83.4%; I^2^ = 87.4%). Regimens with only one antibiotic (35 arms, 6,872 patients) had a rate of 83.8% (95% CI: 81.2 to 86.2%; I^2^ = 86.6%).

##### 3.4.2.8 Antibiotic classes

*β*-lactams: Regimens containing β-lactams (108 arms, 17,358 patients) had an eradication rate of 82.5% (95% CI: 80.7 to 84.2%; I^2^ = 88.7%), similar to regimens without β-lactams (18 arms, 2,583 patients; rate 83.3, 95% CI: 81.0 to 85.4%; I^2^ = 50.3%). Notably, heterogeneity was relatively lower for non-β-lactam regimens.

Macrolides: Regimens containing macrolides (49 arms, 7,997 patients) showed an eradication rate of 82.4% (95% CI: 79.1 to 85.3%; I^2^ = 91.8%), comparable to regimens without macrolides (77 arms, 11,944 patients; rate 82.8, 95% CI: 81.1 to 84.3%; I^2^ = 80.3%).

Fluoroquinolones: Regimens containing fluoroquinolones (12 arms, 1,137 patients) demonstrated a rate of 79.1% (95% CI: 73.3 to 83.8%; I^2^ = 77.8%), slightly lower than regimens without fluoroquinolones (114 arms, 18,804 patients; rate 83.0, 95% CI: 81.3 to 84.6%; I^2^ = 87.8%).

Tetracyclines: Regimens containing tetracyclines (26 arms, 3,580 patients) had an eradication rate of 83.3% (95% CI: 81.3 to 85.1%; I^2^ = 55.4%), similar to regimens without tetracyclines (100 arms, 16,361 patients; rate 82.5, 95% CI: 80.5 to 84.3%; I^2^ = 89.2%). Heterogeneity was notably lower for tetracycline-containing regimens.

Nitroimidazoles: Regimens containing nitroimidazoles (32 arms, 4,249 patients) showed a higher eradication rate of 84.8% (95% CI: 82.4 to 86.9%; I^2^ = 73.8%) compared to regimens without nitroimidazoles (94 arms, 15,692 patients; rate 81.9, 95% CI: 79.9 to 83.7%; I^2^ = 89.0%).

#### 3.4.3 Meta-regression of overall eradication rate

Meta-regression analyses were conducted with eradication rate as the outcome variable (Table 3). Publication year, continent distribution, study type, and acid suppressant type emerged as significant factors explaining heterogeneity in eradication rates. In contrast, regimen-specific characteristics such as dosage intensity, number of antibiotics, and antibiotic classes did not demonstrate statistical significance in the regression models.

**Table 3:** Meta-regression results of eradication rates.

Covariate	Coefficient (β)	95% CI	*P*-value	R^2^
Dose_intensity	0.0060	[−0.2430, 0.2550]	0.9621	0.000
Scheme_type	0.0065	[−0.1026, 0.1156]	0.9063	0.000
Bismuth_compound	0.1403	[−0.0704, 0.3509]	0.1899	0.014
Acid_suppression_type	0.3527	[0.1220, 0.5835]	0.0030	0.069
Treatment_plan_course	0.0067	[−0.0383, 0.0517]	0.7697	0.001
The_quantity_of_antibiotics	−0.0487	[−0.2391, 0.1416]	0.6131	0.002
β-lactam_antibiotics	−0.1415	[−0.4524, 0.1693]	0.3693	0.007
Macrolide_antibiotics	−0.1452	[−0.3579, 0.0674]	0.1789	0.015
Quinolones_antibiotics	−0.1992	[−0.6207, 0.2223]	0.3514	0.007
Tetracyclines_antibiotics	0.1355	[−0.1363, 0.4072]	0.3256	0.008
Nitroimidazoles_antibiotics	0.2152	[−0.0471, 0.4774]	0.1069	0.021
Population_characteristics	−0.0757	[−0.3305, 0.1792]	0.5578	0.003
Publication_year	0.0411	[0.0224, 0.0599]	0.0000	0.132
Continent	−0.1945	[−0.2870, −0.1020]	0.0001	0.123
Research_type	−0.3749	[−0.6342, −0.1157]	0.0049	0.062

Publication Year: Eradication rates showed a significant increasing trend over time (*β* = 0.0411, 95% CI: 0.0224 to 0.0599, *p* < 0.0001, R^2^ = 13.2%). This suggests that more recent studies report higher eradication rates, potentially reflecting improvements in regimen optimization and antimicrobial resistance management.

Continent Distribution: Geographic location significantly influenced eradication rates (*β* = −0.1945, 95% CI: −0.2870 to −0.1020, *p* = 0.0001, R^2^ = 12.3%), reflecting systematic differences in regional resistance patterns, treatment strategies, and population characteristics.

Study Type: Study design was also associated with eradication rates (*β* = −0.3749, 95% CI: −0.6342 to −0.1157, *p* = 0.0049, R^2^ = 6.2%), indicating differences in reported eradication rates between randomized and non-randomized studies.

Acid Suppressant Type: The type of acid suppressant significantly affected eradication rates (*β* = 0.3527, 95% CI: 0.1220 to 0.5835, *p* = 0.0030, R^2^ = 6.9%), potentially attributable to the more potent acid-inhibitory effect of PCABs.

Other Factors: Variables including dosage intensity, regimen type, bismuth use, treatment duration, number of antibiotics, presence of specific antibiotic classes (β-lactams, macrolides, fluoroquinolones, tetracyclines, nitroimidazoles), and population characteristics all yielded *p*-values > 0.05 with R^2^ values ranging from 0 to 2.1%, indicating no statistically significant contribution to explaining heterogeneity.

#### 3.4.4 Sensitivity analysis of overall eradication rate

Sensitivity analysis using the leave-one-out method was performed to assess the robustness of the pooled estimates. In the primary analysis including all studies, sequential exclusion of any single study or treatment arm yielded pooled eradication rates consistently ranging from 82.8 to 83.2%, closely approximating the original pooled rate of 82.99%. All resulting 95% confidence intervals overlapped with the original interval (81.4–84.5%), indicating no significant deviation. The I^2^ statistic for heterogeneity remained consistently high, ranging from 86.8 to 87.4%, suggesting that the between-study heterogeneity was not driven by any particular study.

To further evaluate the impact of study design on the pooled effect size, a sensitivity analysis was conducted restricted to randomized controlled trials. In this analysis, the pooled eradication rate was 81.6% (95% CI: 79.7 to 83.4%), which was closely aligned with the primary analysis result (82.99%), differing by approximately 1.4 percentage points. Leave-one-out sensitivity analysis within the RCT subset demonstrated that exclusion of any single RCT yielded pooled eradication rates stable between 81.4 and 81.8%, with minimal fluctuation, indicating robust results within this subgroup as well. The I^2^ statistic remained high (86.3–87.0%), similar to the heterogeneity level observed in the primary analysis, suggesting that the substantial between-study heterogeneity primarily stemmed from differences in treatment regimens themselves rather than study design variations.

These findings indicate that the overall efficacy estimates are not dependent on any single study, regardless of whether non-randomized studies are included, supporting the robustness of the pooled results from this meta-analysis. The high consistency between the RCT-only analysis and the primary analysis further substantiates the reliability of the study conclusions.

### 3.5 Analysis of overall adverse events

#### 3.5.1 Meta-analysis of overall adverse event incidence and publication Bias assessment

**Figure 6:**
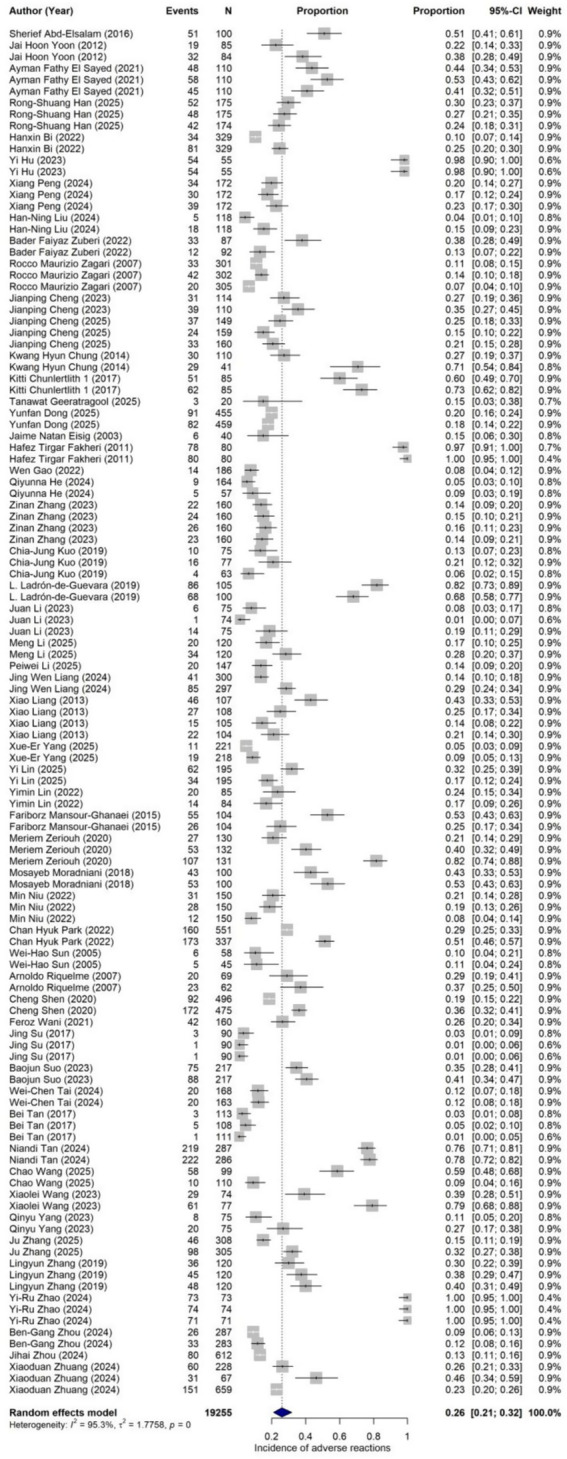
Forest plot of adverse reactions.

Adverse event data were extracted from 54 studies, encompassing 119 treatment arms with a total of 19,255 participants, among whom 5,202 experienced adverse events of varying severity. The forest plot for overall adverse event incidence is presented in Figure 6. The random-effects model (employing Hartung-Knapp correction) yielded a pooled overall adverse event incidence of 26.09% (95% CI: 21.03 to 31.87%) for *H. pylori* eradication therapy. Extremely high heterogeneity was observed across studies (I^2^ = 95.3%; τ^2^ = 1.7758; Q = 2495.03, df = 118, *p* < 0.0001).

**Figure 7:**
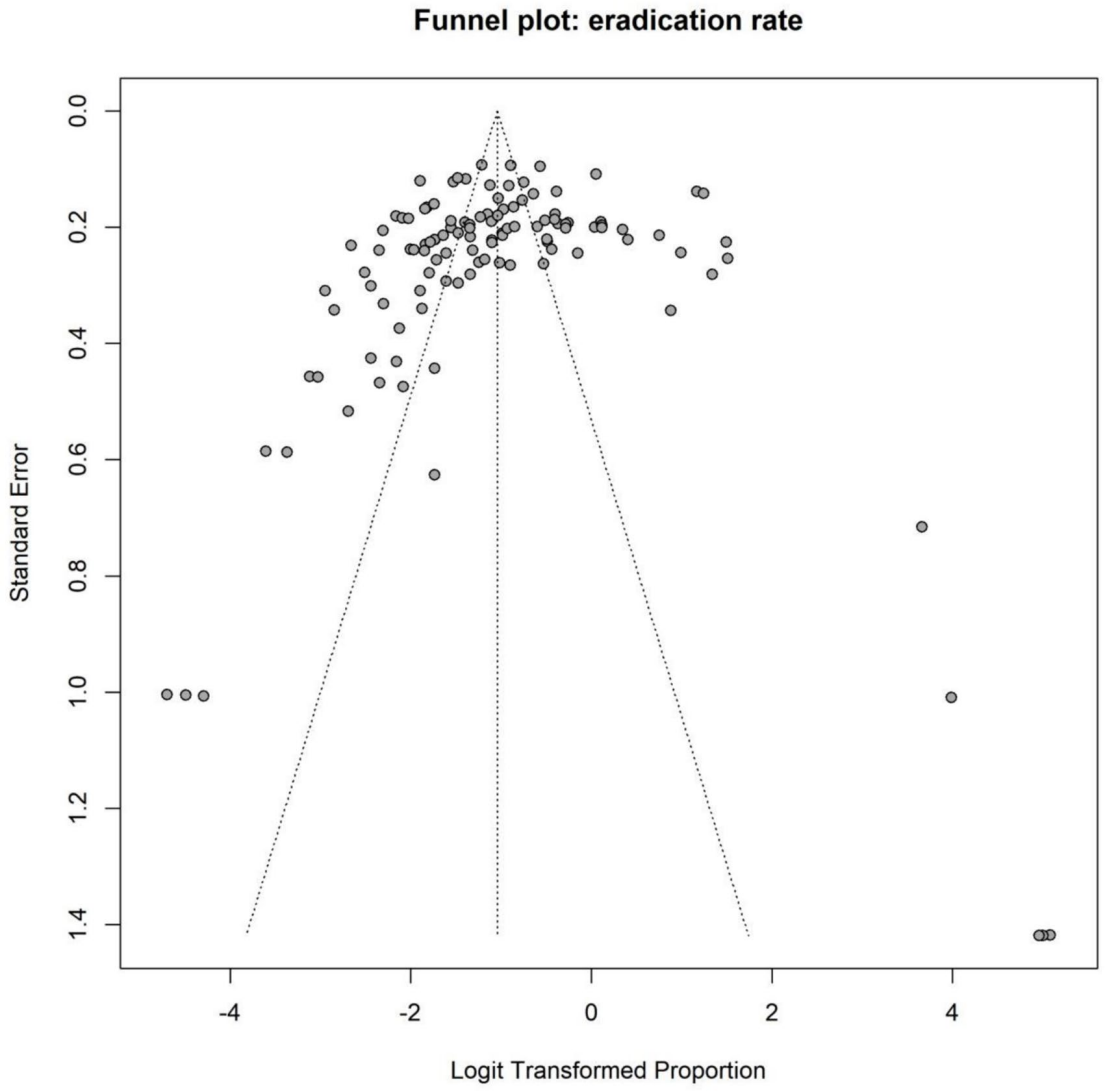
Funnel plot of adverse reactions.

Regarding publication bias, neither Egger’s test (*t* = −1.18, df = 117, *p* = 0.2417) nor Begg’s test (*z* = −1.39, *p* = 0.1633) reached statistical significance, suggesting that the pooled estimate for adverse event incidence was not substantially influenced by publication bias. The consistency between these two tests indicates that the safety-related conclusions of this study are relatively reliable. However, visual inspection of the funnel plot for overall adverse event incidence (Figure 7) revealed some degree of asymmetry: small studies were distributed at both extremes of the funnel plot, encompassing arms with both low and high adverse event incidence, resulting in an uneven funnel shape. The distribution of medium-sized studies also failed to form a symmetrical inverted funnel. Taken together with the non-significant Egger’s and Begg’s test results, this asymmetry is more likely to reflect genuine between-study heterogeneity rather than systematic publication bias.

#### 3.5.2 Subgroup analysis of overall adverse event incidence

Subgroup analyses of overall adverse event incidence demonstrated that rates across most subgroups ranged from 15 to 40%. However, nearly all subgroups exhibited high to extremely high heterogeneity (I^2^ range: 88–97%), indicating substantial differences across studies in terms of populations, medications, and reporting standards (see Table 4 for details).

**Table 4:** The subgroup analysis results of adverse reactions.

Analysis		Studies	Patients	Pooled rate	95% CI	I^2^	Tau^2^	*Q*	*p*-value	Prediction interval
Dose_intensity	Dose_intensity_standard	94	14,700.0	0.288	[0.250, 0.330]	95.4%	0.8240	2006.01	0.0000	[0.000, 1.000]
	Dose_intensity_high	25	4,555.0	0.159	[0.122, 0.205]	92.0%	0.5229	300.06	0.0000	[0.000, 1.000]
Scheme_type	Scheme_type_triple	28	2,820.0	0.205	[0.142, 0.287]	94.5%	1.2768	490.43	0.0000	[0.000, 1.000]
	Scheme_type_sequential	8	872.0	0.541	[0.410, 0.666]	90.1%	0.4498	70.59	0.0000	[0.000, 1.000]
	Scheme_type_quadruple	50	8,742.0	0.316	[0.263, 0.373]	95.8%	0.7573	1161.97	0.0000	[0.000, 1.000]
	Scheme_type_dual	33	6,821.0	0.158	[0.133, 0.187]	88.0%	0.2873	267.74	0.0000	[0.000, 1.000]
Population_characteristics	Population_characteristics_prior_failure	14	2,334.0	0.292	[0.221, 0.376]	93.0%	0.4570	186.90	0.0000	[0.000, 1.000]
	Population_characteristics_general	102	16,651.0	0.263	[0.227, 0.301]	95.5%	0.8761	2262.94	0.0000	[0.000, 1.000]
	Population_characteristics_symptomatic	3	270.0	0.021	[0.009, 0.050]	0.0%	0.0000	1.48	0.4775	[0.009, 0.050]
Bismuth_compound	Bismuth_compound_no	71	10,998.0	0.233	[0.192, 0.281]	95.7%	1.0306	1642.92	0.0000	[0.000, 1.000]
	Bismuth_compound_yes	48	8,257.0	0.289	[0.243, 0.340]	94.4%	0.6012	843.35	0.0000	[0.000, 1.000]
Acid_suppression	Acid_suppression_type_PPI	86	13,199.0	0.276	[0.238, 0.318]	95.0%	0.7859	1694.05	0.0000	[0.000, 1.000]
	Acid_suppression_type_PCAB	33	6,056.0	0.211	[0.160, 0.273]	95.7%	0.8824	742.33	0.0000	[0.000, 1.000]
Treatment_plan	Treatment_plan_course_14_days	87	15,156.0	0.269	[0.231, 0.311]	95.6%	0.8370	1966.76	0.0000	[0.000, 1.000]
	Treatment_plan_course_7_days	21	1,832.0	0.196	[0.139, 0.270]	90.6%	0.7859	212.37	0.0000	[0.000, 1.000]
	Treatment_plan_course_10_days	11	2,267.0	0.281	[0.181, 0.409]	96.8%	0.8947	309.58	0.0000	[0.000, 1.000]
The_quantity_of_antibiotics	The_quantity_of_antibiotics_3_antibiotics	10	1,346.0	0.526	[0.411, 0.638]	93.1%	0.4977	131.17	0.0000	[0.000, 1.000]
	The_quantity_of_antibiotics_2_antibiotics	74	11,037.0	0.276	[0.234, 0.322]	95.1%	0.8286	1503.79	0.0000	[0.000, 1.000]
	The_quantity_of_antibiotics_1_antibiotic	35	6,872.0	0.161	[0.134, 0.192]	89.2%	0.3376	315.90	0.0000	[0.000, 1.000]
β-lactam_antibiotics	β-lactam_antibiotics_no_β-lactam	16	2,433.0	0.310	[0.256, 0.370]	88.2%	0.2505	127.00	0.0000	[0.000, 1.000]
	β-lactam_antibiotics_with_β-lactam	103	16,822.0	0.249	[0.214, 0.288]	95.7%	0.9322	2346.57	0.0000	[0.000, 1.000]
Macrolide_antibiotics	Macrolide_antibiotics_no_macrolide	72	11,537.0	0.229	[0.197, 0.265]	92.8%	0.5685	984.09	0.0000	[0.000, 1.000]
	Macrolide_antibiotics_with_macrolide	47	7,718.0	0.290	[0.233, 0.355]	96.4%	1.0034	1294.44	0.0000	[0.000, 1.000]
Quinolones_antibiotics	Quinolones_antibiotics_with_quinolone	9	880.0	0.289	[0.184, 0.424]	91.3%	0.6402	91.45	0.0000	[0.000, 1.000]
	Quinolones_antibiotics_no_quinolone	110	18,375.0	0.255	[0.223, 0.291]	95.4%	0.8203	2356.79	0.0000	[0.000, 1.000]
Tetracyclines_antibiotics	Tetracyclines_antibiotics_with_tetracycline	24	3,430.0	0.310	[0.251, 0.375]	91.3%	0.4312	264.79	0.0000	[0.000, 1.000]
	Tetracyclines_antibiotics_no_tetracycline	95	15,825.0	0.241	[0.205, 0.280]	95.8%	0.9258	2220.10	0.0000	[0.000, 1.000]
Nitroimidazoles_antibiotics	Nitroimidazoles_antibiotics_with_nitroimidazole	29	4,060.0	0.371	[0.308, 0.438]	93.3%	0.5153	415.92	0.0000	[0.000, 1.000]
	Nitroimidazoles_antibiotics_no_nitroimidazole	90	15,195.0	0.223	[0.189, 0.260]	95.3%	0.8658	1908.33	0.0000	[0.000, 1.000]

##### 3.5.2.1 Impact of regimen type and structure

Adverse event incidence varied considerably across different regimen types. Sequential therapy showed the highest incidence (54.1, 95% CI: 41.0 to 66.6%; I^2^ = 90.1%), whereas dual therapy exhibited the lowest (15.8, 95% CI: 13.3 to 18.7%; I^2^ = 88.0%). Triple and quadruple therapies demonstrated incidences of 20.5% (95% CI: 14.2 to 28.7%; I^2^ = 94.5%) and 31.6% (95% CI: 26.3 to 37.3%; I^2^ = 95.8%), respectively. The number of antibiotics emerged as a clear risk factor: regimens containing three antibiotics had the highest incidence (52.6, 95% CI: 41.1 to 63.8%; I^2^ = 93.1%), significantly higher than regimens containing two antibiotics (27.6, 95% CI: 23.4 to 32.2%; I^2^ = 95.1%) or one antibiotic (16.1, 95% CI: 13.4 to 19.2%; I^2^ = 89.2%).

##### 3.5.2.2 Impact of key antibiotic classes

Specific antibiotic classes were associated with significantly increased adverse event risk. Regimens containing nitroimidazoles demonstrated the highest incidence (37.1, 95% CI: 30.8 to 43.8%; I^2^ = 93.3%). Regimens containing fluoroquinolones (28.9, 95% CI: 18.4 to 42.4%; I^2^ = 91.3%) and macrolides (29.0, 95% CI: 23.3 to 35.5%; I^2^ = 96.4%) also showed markedly elevated risk. In contrast, regimens without these antibiotic classes exhibited relatively lower incidences: regimens without *β*-lactams had an incidence of 31.0% (95% CI: 25.6 to 37.0%; I^2^ = 88.2%), while β-lactam-containing regimens showed an incidence of 24.9% (95% CI: 21.4 to 28.8%; I^2^ = 95.7%).

##### 3.5.2.3 Impact of dosage intensity, treatment duration, and other parameters

Dosage Intensity: Standard-dose regimens demonstrated a higher adverse event incidence (28.8, 95% CI: 25.0 to 33.0%; I^2^ = 95.4%) compared to high-dose regimens (15.9, 95% CI: 12.2 to 20.5%; I^2^ = 92.0%).

Treatment Duration: Seven-day regimens showed the lowest incidence (19.6, 95% CI: 13.9 to 27.0%; I^2^ = 90.6%), while 10-day and 14-day regimens exhibited incidences of 28.1% (95% CI: 18.1 to 40.9%; I^2^ = 96.8%) and 26.9% (95% CI: 23.1 to 31.1%; I^2^ = 95.6%), respectively.

Acid Suppression Type and Bismuth Use: PCAB-based regimens demonstrated a lower incidence (21.1, 95% CI: 16.0 to 27.3%; I^2^ = 95.7%) compared to PPI-based regimens (27.6, 95% CI: 23.8 to 31.8%; I^2^ = 95.0%). Bismuth-containing regimens showed a higher incidence (28.9, 95% CI: 24.3 to 34.0%; I^2^ = 94.4%) compared to non-bismuth regimens (23.3, 95% CI: 19.2 to 28.1%; I^2^ = 95.7%).

##### 3.5.2.4 Impact of population characteristics

Adverse event incidences were comparable between the general population (26.3, 95% CI: 22.7 to 30.1%; I^2^ = 95.5%) and populations with prior eradication failure (29.2, 95% CI: 22.1 to 37.6%; I^2^ = 93.0%). The incidence in exclusively symptomatic populations was extremely low (2.1, 95% CI: 0.9 to 5.0%; I^2^ = 0%); however, this finding is based on a limited number of studies (3 arms) and should be interpreted with caution.

#### 3.5.3 Meta-regression of adverse event incidence

Univariate meta-regression analyses were performed with adverse event incidence as the outcome variable (Table 5). Dosage intensity, regimen type, number of antibiotics, and the use of macrolides or nitroimidazoles demonstrated significant associations with adverse event incidence.

**Table 5:** Meta-regression of adverse reactions.

Covariate	Coefficient (β)	95% CI	P-value	R^2^
Dose_intensity	−0.6476	[−1.0558, −0.2395]	0.0021	0.077
Scheme_type	0.2163	[0.0547, 0.3780]	0.0091	0.056
Bismuth_compound	0.0790	[−0.2577, 0.4157]	0.6430	0.002
Acid_suppression_type	−0.3227	[−0.6900, 0.0446]	0.0845	0.025
Treatment_plan_course	−0.0017	[−0.0757, 0.0722]	0.9628	0.000
The_quantity_of_antibiotics	0.7283	[0.4796, 0.9770]	0.0000	0.220
β-lactam_antibiotics	−0.2114	[−0.6640, 0.2412]	0.3569	0.007
Macrolide_antibiotics	0.5098	[0.1838, 0.8358]	0.0024	0.075
Quinolones_antibiotics	0.6752	[−0.0053, 1.3557]	0.0518	0.031
Tetracyclines_antibiotics	0.1262	[−0.2926, 0.5451]	0.5518	0.003
Nitroimidazoles_antibiotics	0.5422	[0.1758, 0.9086]	0.0041	0.067
Population_characteristics	−0.0065	[−0.4741, 0.4612]	0.9781	0.000
Publication_year	−0.0092	[−0.0472, 0.0289]	0.6346	0.002
Continent	0.0613	[−0.1243, 0.2469]	0.5145	0.004
Research_type	0.1475	[−0.2430, 0.5380]	0.4559	0.005

Dosage Intensity: Dosage intensity was a significant factor influencing adverse events (*β* = −0.6476, 95% CI: −1.0558 to −0.2395, *p* = 0.0021, R^2^ = 7.7%). Under the coding scheme employed in this study, systematic differences in adverse event incidence were observed between dosage intensity categories, with high-dose regimens demonstrating significantly lower adverse event incidence compared to standard-dose regimens.

Regimen Type: Regimen type was significantly associated with adverse events (*β* = 0.2163, 95% CI: 0.0547 to 0.3780, *p* = 0.0091, R^2^ = 5.6%), indicating differences in adverse event incidence across different treatment strategies (e.g., triple, quadruple, sequential therapy).

Number of Antibiotics: The number of antibiotics was the strongest covariate explaining heterogeneity (*β* = 0.7283, 95% CI: 0.4796 to 0.9770, *p* < 0.0001, R^2^ = 22.0%). A greater number of antibiotics was associated with a higher risk of adverse events, with this variable alone explaining 22% of the between-study variance.

Macrolide Use: Use of macrolides was significantly associated with higher adverse event incidence (*β* = 0.5098, 95% CI: 0.1838 to 0.8358, *p* = 0.0024, R^2^ = 7.5%).

Nitroimidazole Use: Use of nitroimidazoles was similarly associated with higher adverse event incidence (β = 0.5422, 95% CI: 0.1758 to 0.9086, *p* = 0.0041, R^2^ = 6.7%).

Fluoroquinolone Use: A marginal trend was observed for fluoroquinolone use (β = 0.6752, *p* = 0.0518), but this did not reach statistical significance.

Acid Suppressant Type: A marginal trend was noted for acid suppressant type (β = −0.3227, *p* = 0.0845, R^2^ = 2.5%), but this was not statistically significant.

Other Covariates: No significant associations were observed for other covariates, including bismuth use, treatment duration, β-lactam use, tetracycline use, population characteristics, publication year, continent, or study type (all *p* > 0.05). R^2^ values for these variables were generally in the 0–2% range, indicating limited explanatory power for between-study heterogeneity.

#### 3.5.4 Sensitivity analysis of adverse event incidence

Sensitivity analysis using the leave-one-out method was performed to assess the robustness of the pooled adverse event incidence estimates. Results demonstrated that sequential exclusion of any single study or treatment arm yielded pooled adverse event incidences ranging from 25.84 to 26.71% (original pooled incidence: 26.09%), representing minimal fluctuation (<0.9 percentage points). The corresponding 95% confidence intervals remained highly stable (lower bounds approximately 22.6–23.4%, upper bounds approximately 29.3–30.2%), all overlapping substantially with the original interval (21.03–31.87%). These findings indicate robust estimation of the overall adverse event rate. Regarding heterogeneity, τ^2^ values remained stable between 0.77 and 0.87 across iterations, and I^2^ consistently remained at extremely high levels (94.96–95.43%), with marginal decreases (<0.5%) observed only in rare instances of study exclusion. This suggests that the substantial between-study heterogeneity was not driven by any particular study but rather reflects systematic differences across studies (e.g., population characteristics, treatment regimens, drug dosages).

To further evaluate the impact of study design on pooled adverse event estimates, a sensitivity analysis was conducted restricted to randomized controlled trials. In this analysis, the pooled adverse event incidence ranged from approximately 25.1 to 25.9% (original pooled estimate within this subset approximately 25.5%), closely aligning with the primary analysis result (26.09%). Leave-one-out sensitivity analysis within the RCT subset demonstrated that exclusion of any single RCT yielded pooled adverse event incidences stable between 24.8 and 25.9%, with minimal fluctuation (<1.1 percentage points), and all resulting 95% confidence intervals showed substantial overlap with the original interval. These findings indicate robust results within the RCT subset as well. Regarding heterogeneity, I^2^ values consistently remained at extremely high levels (95.0–95.6%) across iterations, similar to the heterogeneity observed in the primary analysis, further confirming that the substantial between-study heterogeneity primarily stems from differences in treatment regimens themselves rather than study design variations.

In summary, leave-one-out sensitivity analyses demonstrate that the meta-analysis results for adverse events are robust and reliable, regardless of whether non-randomized studies are included. The main conclusions are not sensitive to any single study, and no substantive changes occur upon exclusion of individual studies. The high consistency between the RCT-only analysis and the primary analysis further substantiates the reliability of the study conclusions.

## 4 Discussion

*Helicobacter pylori* infection represents a significant public health concern, with chronic colonization associated with serious complications including chronic gastritis, peptic ulcer disease, gastric mucosa-associated lymphoid tissue lymphoma, and gastric cancer. Approximately 1–3% of infected individuals may ultimately develop gastric cancer (25–28). Therefore, optimizing both the efficacy and safety of eradication therapy is of paramount importance for disease prevention and control.

### 4.1 Efficacy analysis

The findings of this study indicate that sequential therapy, quadruple therapy, selected dual therapies, PCAB-based regimens, 10-day treatment duration, regimens containing three antibiotics, and nitroimidazole-containing regimens demonstrated relatively higher eradication rates. However, these subgroup analysis results should be considered hypothesis-generating rather than direct guidance for clinical practice, as they were not adjusted for multiple comparisons and could not fully control for confounding factors. Meta-regression suggested that publication year, continental distribution, study design, and acid suppressant type were the primary factors contributing to heterogeneity in eradication rates.

The generally superior eradication rates observed for sequential, quadruple, and selected dual therapies compared to traditional triple therapy align closely with pharmacological mechanisms and resistance patterns. In this study, triple therapy predominantly comprised classic clarithromycin-based regimens, for which clarithromycin resistance constitutes the primary cause of treatment failure (28, 29). When resistance rates exceed 15%, the eradication rate of triple therapy frequently falls below 75% (30, 31). In contrast, sequential therapy, through initial amoxicillin administration that disrupts cell wall integrity and potentially reduces efflux pump activity, may enhance subsequent susceptibility of resistant strains to later antibiotics (32). Bismuth-containing quadruple therapy leverages the multifaceted actions of bismuth, including cell wall disruption, adhesion inhibition, and biofilm interference, maintaining activity against clarithromycin- or metronidazole-resistant strains and thereby sustaining favorable efficacy across diverse resistance settings (33). The dual therapy regimens demonstrating favorable performance in this study were predominantly based on high-dose amoxicillin combined with potent acid suppressants (e.g., PCABs). This approach extends the duration of amoxicillin bactericidal activity through enhanced acid suppression, thereby improving eradication efficiency (30, 34).

Regional antimicrobial resistance patterns likely constitute a critical determinant of observed efficacy variations. The majority of studies included in this meta-analysis originated from East Asia. A 2024 systematic review focusing on the Asia-Pacific region published in The Lancet by Hong et al. reported that, as of 2022, primary clarithromycin resistance in this region reached 30% (95% CI 28–33), metronidazole resistance reached 61% (95% CI 55–66), and both had been increasing continuously since 1990, substantially exceeding the 15% threshold associated with triple therapy failure (35). East Asia, as a region with high antimicrobial resistance within the Asia-Pacific, may exhibit suboptimal efficacy of regimens effective in lower-resistance settings (e.g., classic triple therapy) (36). Consequently, the observed differences in efficacy may partially reflect regional variations in resistance backgrounds rather than being determined solely by regimen structure.

Meta-regression further elucidated key factors influencing efficacy. The significant effect of publication year reflects the evolution of clinical practice over time, including the widespread adoption of potent acid suppressants, optimization of antibiotic combinations, and maturation of rescue treatment strategies (37). The influence of continental distribution reflects differences in regional resistance profiles, with high resistance rates in East Asia directly impacting regimen efficacy (38, 39). The impact of study design relates to factors including patient selection, adherence to treatment protocols, and completeness of follow-up (40). The effect of acid suppressant type derives from differences among these agents in terms of intragastric duration and stability.

### 4.2 Safety analysis

Regarding safety, the pooled incidence of adverse events was 26.09%, with the majority being mild to moderate in severity. Severe adverse events (1.2%) and intolerable reactions (0.3%) were uncommon. Sequential therapy demonstrated the highest adverse event incidence; regimens containing three antibiotics, macrolides, or nitroimidazoles were also associated with more frequent adverse events. Meta-regression identified dosage intensity, regimen type, number of antibiotics, and specific antibiotic classes (macrolides, nitroimidazoles) as significant contributing factors, with the number of antibiotics alone explaining approximately 22% of the observed heterogeneity. It should be noted that these safety findings are likewise derived from subgroup analyses and should be considered exploratory in nature.

The highest adverse event rate observed with sequential therapy may stem from its two-phase, multi-drug design, which exposes patients to different antibiotics in succession over a short period, potentially leading to cumulative side effects (41). The frequent adverse events associated with regimens containing three antibiotics are likely attributable to the increased cumulative dose burden, greater disruption of the intestinal microbiota, and potential drug–drug interactions (e.g., effects on metabolic enzymes) (42). Regimens combining macrolides or nitroimidazoles demonstrated poorer tolerability, primarily related to the potent direct gastrointestinal irritant effects of these drug classes (43, 44).

### 4.3 Limitations

This study has several limitations. First, the included treatment regimens were highly heterogeneous, exhibiting substantial variability in antibiotic combinations, dosage intensity, treatment duration, and type of acid suppressant. This resulted in extremely high heterogeneity in both overall and subgroup analyses (I^2^ > 85% for most analyses), which could not be fully explained by the subgroup analyses and meta-regression performed. Therefore, the pooled effect sizes from this study should be interpreted with caution, reflecting overall trends in the available evidence rather than providing precise effect estimates. Future research employing more homogeneous study designs and further exploration of potential sources of heterogeneity are warranted.

Second, definitions and reporting methods for adverse events varied considerably across studies, with some failing to report severity grading. This inconsistency may have introduced bias into the pooled analyses or led to underestimation of risk. Establishment of standardized adverse event reporting systems is needed in future research.

Third, the inclusion of some non-randomized studies may have introduced selection bias and confounding factors, potentially affecting the accuracy of effect estimates.

Fourth, there was marked geographic imbalance in the distribution of included studies, with the majority originating from the Asia-Pacific region, particularly China. Given the substantial regional variations in *H. pylori* resistance patterns (e.g., clarithromycin resistance rates are generally lower in Europe and North America compared to Asia), extrapolation of these findings to regions with different resistance profiles requires considerable caution. Future high-quality studies from Europe, North America, and other regions are encouraged to validate the generalizability of these findings globally.

Fifth, this study included multiple treatment arms from multiple arms of the study as independent data points. Although we conducted a sensitivity analysis to assess the impact of individual studies on the combined effect size, and methodologically clarified that this is a common approach in single-arm rate meta-analysis, there may be inherent correlations among multiple treatment arms from the same study (such as sharing the same study population characteristics, research centers, and research periods). This non-independence cannot be completely eliminated statistically and may have a certain impact on the estimation of the standard error of the effect size.

## 5 Conclusion

This study systematically evaluated the efficacy and safety of various *Helicobacter pylori* eradication regimens, revealing substantial differences among treatment protocols in terms of both eradication rates and adverse event profiles. Within the available evidence, sequential therapy, quadruple therapy, and selected dual therapies demonstrated trends toward higher eradication rates, whereas the efficacy of triple therapy appeared more susceptible to the influence of antimicrobial resistance patterns. PCAB-based regimens and high-dose amoxicillin combination therapies suggested potentially greater efficacy stability across multiple analyses. Regarding safety, sequential therapy, regimens containing three antibiotics, and those including macrolides or nitroimidazoles were associated with higher adverse event incidence, indicating that a balance must be struck between efficacy and tolerability in clinical decision-making.

It is important to emphasize that these findings are exploratory in nature, derived primarily from descriptive and associative analyses. They are intended to characterize the performance profiles of different treatment regimens within the existing literature and to inform clinical decision-making by providing directional references, rather than to establish definitive treatment priorities. In summary, this study offers a comprehensive evidence base for comparing diverse therapeutic regimens, contributing to the development of more rational, individualized treatment strategies tailored to varying resistance backgrounds and patient characteristics. Future high-quality, large-scale, multicenter studies are warranted to further validate the comparative merits and limitations of different regimens.

## Data Availability

The original contributions presented in the study are included in the article/Supplementary material, further inquiries can be directed to the corresponding author.
