# Supplementary material for: Efficacy and safety of antibiotic regimens for Helicobacter pylori eradication: a systematic review and meta-analysis
Source: Front Med (Lausanne). 2026 Apr 7;13:1780052. doi: 10.3389/fmed.2026.1780052 (PMC13095567; doi:10.3389/fmed.2026.1780052)
Supplement: Supplementary file 1 [file Table_1.DOCX]

| Database | Search expression |
| --- | --- |
| Pubmed | ("Helicobacter pylori"[Mesh] OR "H. pylori" OR Helicobacter[tiab])  AND  (eradication[tiab] OR therapy[tiab] OR treatment[tiab] OR quadruple[tiab] OR triple[tiab]  OR bismuth[tiab] OR PPI[tiab] OR "proton pump inhibitor*"[tiab] OR vonoprazan[tiab] OR PCAB[tiab]  OR amoxicillin[tiab] OR clarithromycin[tiab] OR metronidazole[tiab] OR tetracycline[tiab]  OR levofloxacin[tiab] OR rifabutin[tiab] OR concomitant[tiab] OR sequential[tiab] OR hybrid[tiab] OR HDDT[tiab])  AND  (adverse[tiab] OR "adverse event*"[tiab] OR safety[tiab] OR toxicity[tiab] OR side-effect*[tiab]  OR diarrhea[tiab] OR diarrhoea[tiab] OR nausea[tiab] OR vomiting[tiab] OR abdominal pain[tiab]  OR dysgeusia[tiab] OR rash[tiab] OR photosensit*[tiab] OR hepatotox*[tiab] OR "C. difficile"[tiab])  AND Humans[Mesh]  NOT (Review[pt] OR Meta-Analysis[pt] OR "Systematic Review"[pt] OR "Case Reports"[pt]  OR Editorial[pt] OR Letter[pt] OR Comment[pt])  NOT (animals[mh] NOT humans[mh])  NOT ("in vitro"[tiab] OR "cell line*"[tiab] OR mice[tiab] OR mouse[tiab] OR rat[tiab]) |
| Embase | ('helicobacter pylori'/exp OR 'h pylori':ti,ab OR helicobacter:ti,ab)  AND  (eradication:ti,ab OR therapy:ti,ab OR treatment:ti,ab OR quadruple:ti,ab OR triple:ti,ab  OR bismuth:ti,ab OR ppi:ti,ab OR vonoprazan:ti,ab OR pcab:ti,ab  OR amoxicillin:ti,ab OR clarithromycin:ti,ab OR metronidazole:ti,ab OR tetracycline:ti,ab  OR levofloxacin:ti,ab OR rifabutin:ti,ab OR concomitant:ti,ab OR sequential:ti,ab OR hybrid:ti,ab OR hddt:ti,ab)  AND  ('adverse event*':ti,ab OR safety:ti,ab OR toxicity:ti,ab OR 'side effect*':ti,ab  OR diarrhea:ti,ab OR diarrhoea:ti,ab OR nausea:ti,ab OR vomiting:ti,ab OR 'abdominal pain':ti,ab  OR dysgeusia:ti,ab OR rash:ti,ab OR photosensit*:ti,ab OR hepatotox*:ti,ab OR 'clostridioides difficile':ti,ab)  AND 'human'/exp  NOT ('review'/it OR 'systematic review'/it OR 'meta analysis'/it OR 'case report'/it OR 'conference abstract'/it)  NOT ('animal'/exp NOT 'human'/exp)  NOT ('in vitro study'/exp OR 'animal experiment'/exp) |
| Web of science | TS=("Helicobacter pylori" OR "H. pylori" OR Helicobacter)  AND  TS=(eradication OR therapy OR treatment OR triple OR quadruple OR bismuth OR PPI OR "proton pump inhibitor*"  OR vonoprazan OR PCAB OR amoxicillin OR clarithromycin OR metronidazole OR tetracycline  OR levofloxacin OR rifabutin OR concomitant OR sequential OR hybrid OR HDDT)  AND  TS=("adverse event*" OR "side effect*" OR safety OR toxicity OR diarrhea OR diarrhoea OR nausea OR vomiting  OR "abdominal pain" OR dysgeusia OR rash OR photosensitivity OR hepatotoxicity OR "C. difficile")  NOT  DT=("Review" OR "Meta-Analysis")  NOT  TS=("case report" OR "case series" OR "in vitro" OR "animal model" OR mice OR rat OR rabbit) |

Supplementary Table 1. Summary of Search Terms

| Author (Year) | Choice | Comparability | Exposure / Result | Total score |
| --- | --- | --- | --- | --- |
| Sherief Abd-Elsalam (2016) | 3 | 1 | 3 | 7 |
| Kwang Hyun Chung (2014) | 3 | 1 | 3 | 7 |
| Jaime Natan Eisig (2003) | 3 | 1 | 3 | 7 |
| Wen Gao (2022) | 3 | 1 | 3 | 7 |
| Chia-Jung Kuo (2019) | 3 | 2 | 3 | 8 |
| Peiwei Li (2025) | 3 | 1 | 3 | 7 |
| Jing Wen Liang (2024) | 3 | 1 | 3 | 7 |
| Chan Hyuk Park (2022) | 3 | 2 | 3 | 8 |
| Feroz Wani (2021) | 3 | 1 | 3 | 7 |
| Wei-Chen Tai (2024) | 3 | 1 | 3 | 7 |
| Yi-Ru Zhao (2024) | 3 | 1 | 3 | 7 |
| Jihai Zhou (2024) | 3 | 1 | 3 | 7 |
| Xiaoduan Zhuang (2024) | 3 | 2 | 3 | 8 |

Supplementary Table2. Risk of Bias Table for Non-Randomized Controlled Studies


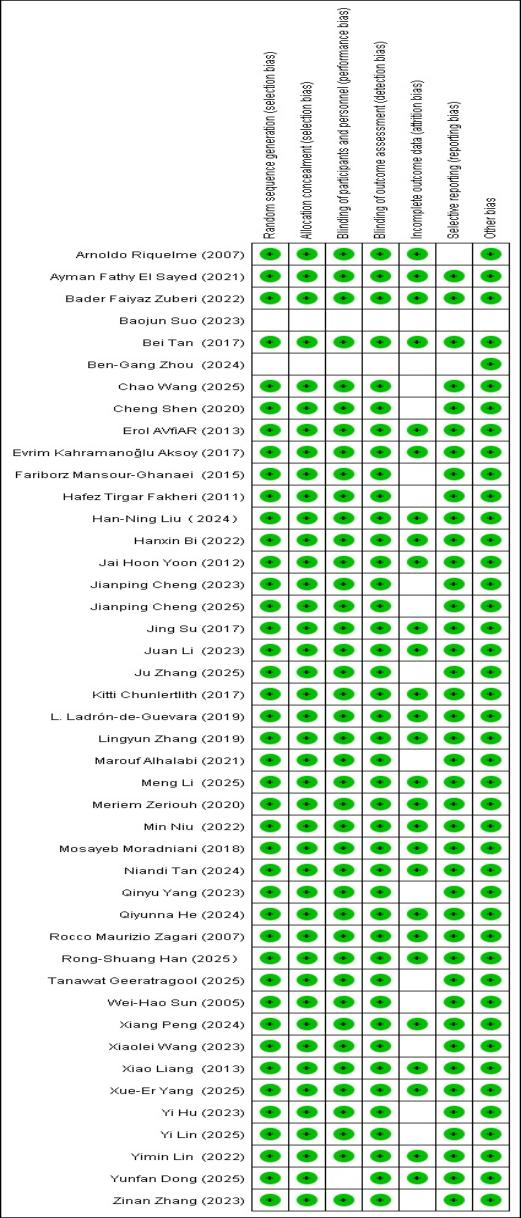


Supplementary Figure 1.Risk of bias summary


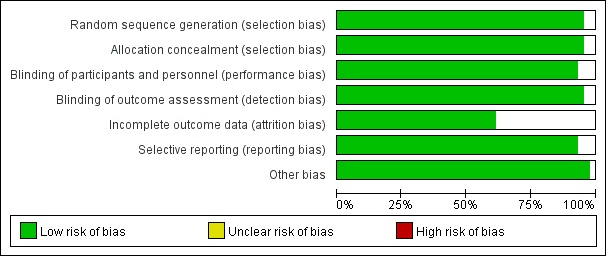


Supplementary Figure 2.Risk of bias graph
